# A Review of Low-Cost Particulate Matter Sensors from the Developers’ Perspectives

**DOI:** 10.3390/s20236819

**Published:** 2020-11-29

**Authors:** Brigida Alfano, Luigi Barretta, Antonio Del Giudice, Saverio De Vito, Girolamo Di Francia, Elena Esposito, Fabrizio Formisano, Ettore Massera, Maria Lucia Miglietta, Tiziana Polichetti

**Affiliations:** 1 ENEA CR-Portici, TERIN-FSD Department, P.le E. Fermi 1, 80055 Portici, Italy; brigida.alfano@enea.it (B.A.); antonio.delgiudice@enea.it (A.D.G.); girolamo.difrancia@enea.it (G.D.F.); elena.esposito@enea.it (E.E.); fabrizio.formisano@enea.it (F.F.); ettore.massera@enea.it (E.M.); mara.miglietta@enea.it (M.L.M.); tiziana.polichetti@enea.it (T.P.); 2 Department of Physics, University of Naples Federico II, via Cinthia, 80100 Napoli, Italy; luigi.barretta3@unina.it; 3 STmicroelectronics, via R. De Feo, Arzano, 80022 Napoli, Italy

**Keywords:** particulate matter, low cost particulate matter sensors, IoT AQ nodes, air quality, air quality monitoring, calibration, characterization, performances

## Abstract

The concerns related to particulate matter’s health effects alongside the increasing demands from citizens for more participatory, timely, and diffused air quality monitoring actions have resulted in increasing scientific and industrial interest in low-cost particulate matter sensors (LCPMS). In the present paper, we discuss 50 LCPMS models, a number that is particularly meaningful when compared to the much smaller number of models described in other recent reviews on the same topic. After illustrating the basic definitions related to particulate matter (PM) and its measurements according to international regulations, the device’s operating principle is presented, focusing on a discussion of the several characterization methodologies proposed by various research groups, both in the lab and in the field, along with their possible limitations. We present an extensive review of the LCPMS currently available on the market, their electronic characteristics, and their applications in published literature and from specific tests. Most of the reviewed LCPMS can accurately monitor PM changes in the environment and exhibit good performances with accuracy that, in some conditions, can reach *R*^2^ values up to 0.99. However, such results strongly depend on whether the device is calibrated or not (using a reference method) in the operative environment; if not, *R*^2^ values lower than 0.5 are observed.

## 1. Introduction

The impact of anthropic activities on environmental integrity and their deleterious consequences on human health have pushed more and more citizens, all over the world, to organize and become informed about the quality of the air they breathe; such a change in social attitudes has been greatly accelerated by the internet’s diffusion [1,2]. Solid matter suspended in air, known as particulate matter (PM), is an air pollutant that has sparked the greatest concern among citizens because of its negative effects on health [3,4,5]. More recently, the possible correlation between PM and the diffusion of the COVID-19 pandemic has led, in recent months, to a further increase of interest in this topic [6,7,8].

This change in social attitudes has resulted in challenging tasks both for the national organizations charged to monitor the air quality and for the governmental institutions tasked to define the operational rules to be observed by those organizations [9,10]. At present, air quality (AQ) regulatory monitoring is performed similarly all over the world by means of regulatory stations (RSs). The most relevant problem in this type of architecture is the limited number of deployed RSs, each of which usually represents an area of tens or even hundreds of square kilometers; moreover, in general, RSs provide pollution data only as hourly averages [11,12,13]. Such architecture is, therefore, clearly unable to comply with the citizens’ requests to be promptly and locally informed on the AQ of the air they breathe unless the RS network is better secured and technically improved. 

Indoor AQ monitoring scenarios are potentially even more complex. While for an outdoor environment, there is a shared accepted value for the harmless concentration limits of pollutants, a similar general consensus for indoor areas does not exist [14]. This is worrying, as humans spend less than 20% of their lifetimes in outdoor environments; thus, they are much more likely to be exposed to dangerous pollutants when they are at the workplace or at home. 

The size and cost of RSs make tightening the monitoring network impractical; therefore, the approaches proposed to overcome this difficulty rely mainly on modeling the outdoor air composition using RS data along with traffic and satellite observations as support [15,16]. Unfortunately, urban settlements can be very difficult to model, as they are characterized by complex fluid-dynamics, which can rapidly change due to a modification of meteorological conditions and/or of the poor models available for anthropic activities [17]. 

Several research groups have suggested that a much denser network of monitoring stations equipped with low-cost sensing devices could support the poor granularity of the RS network. These monitoring stations, fixed or mobile and wirelessly connected to both each other and a main server, can act as nodes to support the RS network and provide citizens the information they require [18]. Attention towards such nodes has recently grown due to the increasing availability of low-cost and powerful single board computer platforms (e.g., Arduino, RasberryPi, the STm32 family, etc.). Among other features, the battery power consumption of such computers is no longer a limit for long-term in-field displacement, which has greatly improved the node performance. 

As a result, several research and industrial projects, both public and private, have developed all over the world to investigate the in-field operation of networks using low-cost AQ nodes that often use RSs (or equivalent methods) [19], which are further discussed in the following Section 2 to self-calibrate themselves throughout their operational lifetimes [20,21]. The promising results observed thus far have fostered intense efforts to define the regulatory frame for these nodes, primarily the specifications these nodes have to satisfy to ensure proper data quality objectives (DQO) and to what extent the data produced can be effectively used to improve the RS monitoring network and respond to citizens’ demands [22,23,24,25,26,27]. 

Such low-cost nodes rely on solid-state chemical sensors for gaseous air pollution monitoring, a class of electronic devices that has been investigated and developed since the 1960s [28,29,30]; these sensors also use relatively less popular low-cost PM sensors (LCPMS). This review focuses on this latter class of sensors, which represents a new frontier in research and industrial development, with new products being continuously proposed and investigated (briefly summarized in Table 1). Only seven models were reported in the first review on this topic in 2017 [31], while the recent paper by Morawska [20] reported more than 20 models. In the present review, about 50 models from 19 different manufacturers are recognized. 

For the sake of clarity, we will not discuss PM monitors as defined by McKercher and coworkers [32], rather we will focus on the basic PM sensor devices that can be used to realize IoT AQ nodes.

Here, we review this specific class of PM sensor devices, focusing on devices that are, at present, on the market and are suitable to be integrated into low-cost AQ nodes in a networking scenario. We, therefore, discuss only those devices whose electronic characteristics enable their use in what is commonly referred to as an Internet of Things (IoT) networking frame. For the sake of clarity, we will discuss only the basic PM sensor devices that can be used to realize IoT AQ nodes. 

Section 2 reports on the basic definitions related to PM and its measurements according to international regulations. In Section 3, the device’s operating principles are discussed. Section 4 is devoted to discussing several methodologies used by various laboratories to characterize and calibrate LCPMS and their possible limitations. In Section 5, we present an extensive review of the LCPMS that are now commercially available, as well as their electronic characteristics, highlighting the factors that make such sensors suitable for wireless network applications. Section 6 mainly discusses the physical characteristics of the sensors presented in Section 5 to extract (from published literature or from specific tests) the most relevant device parameters. Finally, Section 7 is devoted to a general discussion and our conclusions. Additional metrological characteristics of the analysed PM sensors are then included in the Supplementary Information (SI) tables.

## 2. Particulate Matter Basics and Measurement Parameters

Ambient air usually contains a certain amount of solid matter that comes from natural sources such as soil, wind-blown dust (aeolian processes), or anthropic activities. In indoor environments, this solid matter can contain anthropogenic and natural biogenic materials (for example, human and animal hair, skin cells, textile fibers, and plant pollen) and many other materials that may be found in the local environment. In the last century, due to large-scale industrial development and the extensive use of fossil fuels, the composition and amount of solid matter suspended in the air have started to change, raising concerns about their impact on human health. As soon as this particulate matter was recognized as a potential threat to public health, many countries were prompted to establish air quality standards. In the US, the Clean Air Act established in 1970 represented a milestone. In this act, standards for six pollutants were set, and, since then, air pollution has been regulated all over the world through a series of laws and regulations [33]. Particulate matter is one of these pollutants and is defined as a suspension of solid, liquid, or a combination of solid and liquid particles in the air, classified according to the size of the particle diameter (measured in micrometers) [34]. The standards in this act were based on the mass of the total suspended material (TSP) and set considering the mortality associated with short-term air pollution episodes. The primary standard included both an annual standard of 75 $\mu g/m^{3}$ and a maximum daily average of 260 $\mu g/m^{3}$, not to be exceeded more than once per year. After the first data related to the influence of particle size on the lung-deposition of inhaled PM had been collected and developed, revision work on PM criteria began. This debate was largely focused on the specific size fraction to use. In 1980, the Council of the European Communities launched Directive 80/779/EEC, but the first clear definition of PM10 dates to the late 1980s when the US Environmental Protection Agency (EPA) developed a new National Ambient Air Quality Standard for the fraction of suspended particulate matter with an aerodynamic size below 10 μm [35] and set the limit values. Ten years later, relying on a continuous review of scientific knowledge about the health and welfare effects of particulate matter, the EPA promulgated a new air quality standard for particulate matter: PM2.5 [36]. In Europe, similar measures were taken in 1996 with Directive 96/62/EC, and monitoring of the PM2.5 fraction then began in 1999 with the first Daughter Directive (1999/30/EC). Analogous to the PM10 definition, PM2.5 is defined as a particle fraction captured with 50% efficiency at 2.5 μm. According to these definitions, Directive 99/30/EC establishes the methodology for the assessment of PM10 concentrations, either through a reference method for sampling and measurement (CEN Standard EN12341(3)) or through the use of instruments with demonstrated equivalence to a reference instrument.

The current standards for PM exposure are summarized in Table 2 along with the WHO guideline values. These last values are generally stricter than the comparable politically agreed-upon EU and US standards.

The limit values for PM10 and PM2.5 are derived from human epidemiological data, using laboratory animal and in vitro data only in a supporting role [37]. 

When exploring health effects, not only the physical properties of particulate matter, such as particle size and shape, but also the chemical properties (e.g., composition, redox capacity, solubility, etc.) must be considered. Indeed, if the particle dimensions determine the degree of penetration within the respiratory system, the chemical characteristics determine the ability of the particles to react with other pollutants, human biological fluids, or target organs. The chemical composition of particulate matter is extremely heterogeneous, and many of its components, such as organic and inorganic compounds, metals, and acids, depend on the source. The average contents of inorganic ions (such as sulfates, nitrates, and ammonium), organic carbon compounds, and elemental carbon undergo significant variation according to the geographical features of the sampling sites and related human activities, as reported by Snider et al. [38]. In Europe, the main sources of PM emissions (both PM10 and PM2.5) are combustion processes, such as fuel combustion (transport) and the combustion used in commercial, institutional, and household heating [11]. This is reflected in the prevalence of black carbon, especially in fine size fractions [39]. Vehicular particulates are, in addition, often coated with condensed organic and inorganic compounds [40], such as the compounds from the use of automobile catalytic converters, which employ metals that may lead to the presence of platinum (Pt), palladium (Pd), and rhodium (Rh) in their emitted particulates [41]. Natural sources of PM, such as volcanic eruptions, are richer in sulfur particles. Overall, each source has a peculiar emission profile with respect to both particle size and chemical composition. In Table 3, a brief summary of the most common sources of PM by categories is reported.

Indoor environments are not excluded from the PM issue. Indeed, a considerable number of works focus on characterizing the indoor sources of particulate matter. Li et al. in [42] report that smoking, cooking, burning fuel for heating, and burning incense are among the most important internal sources. For example, the authors report that the PM values are 287 $\mu g/m^{3}$ and 34 $\mu g/m^{3}$ in bars and pubs where smoking is allowed and where it is forbidden, respectively. Cooking is another primary source of PM in indoor environment. A study by Dacunto et al. [43] demonstrated that, under all the cooking methods used for various foods, the aerodynamic diameter of the median mass (MMD) never exceeds the micron. This means that the majority of the particulate matter produced in a normal kitchen is of a fine or ultra-fine type. The results of a case study of restaurants in Turkey indicated that cooking is a significant source of indoor particulate matter that, at selected restaurants, even resulted in exposure to As and Cr (VI) [44]. Moreover, it was found that a simple operation like cooking popcorn in the microwave can result in PM2.5 emissions of 1900 $\mu g/m^{3}\;$, i.e., over 50 times higher than the national standard of ambient air for PM2.5 [45]. Another source of indoor particulates is environmental home deodorizers (whether combustive, passive, electrical, or spray-based), which were found to produce particles with a diameter less than 2 microns and, therefore, of a fine type that can reach the respiratory tracts [46]. 

### Measurement Techniques

**Figure 1:**
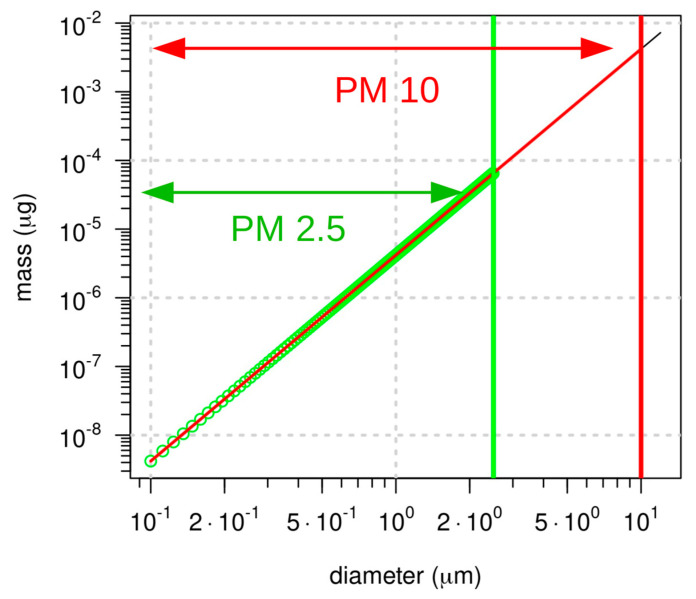
Mass particle vs. diameter for the two classes of particulate (PM2.5 (**green**) and PM10 (**red**)) in a logarithmic scale.

To correctly measure the concentration of PM10 and PM2.5, it is necessary to unambiguously define what is meant by particle size. Irregular shapes usually characterize particles suspended in the air. Consequently, size generally refers to the aerodynamic diameter, i.e., the equivalent diameter of a spherical particle with a density of $1g/{\mathrm{cm}}^{3}$ that has the same settling velocity as the irregular particle. Since PM represents a class of objects with different dimensions and size distributions, when dealing with the total mass of particles below a nominal size, it must be considered that the particle mass grows with the third power of the diameter. Thus, larger particles mostly determine the total measured mass (see Figure 1). 

**Figure 2:**
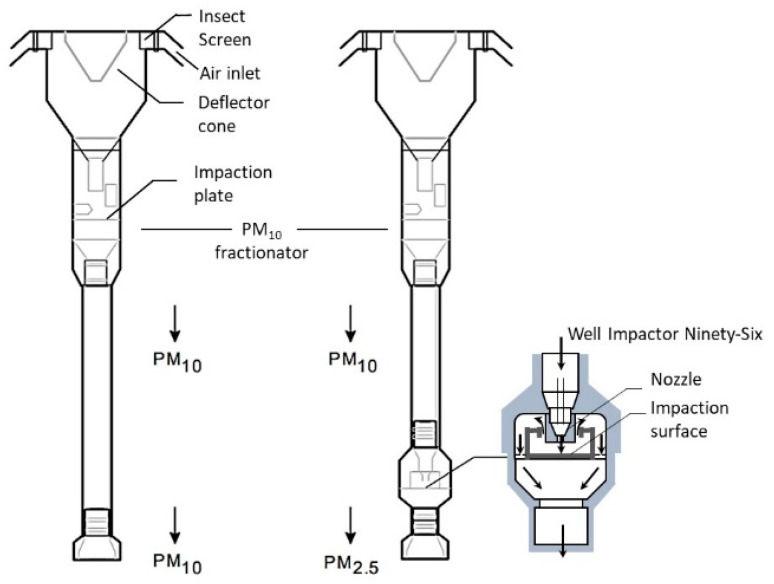
Schematic of a standard low-vol PM10 inlets aspirating at 16.7 lpm (actual conditions). On the right the schematic of a sampling head equipped with PM2.5 aerosol fractionation using a well impactor ninety-six (WINS).

The UNI EN 12341: 2014 and EPA 40 CFR PART 50 standards regulate the reference measuring methodology. This method basically consists of using a sampling head to select the PM according to its size fraction (PM10 or PM2.5), using a filtering system for the collection of particulate matter, followed by gravimetric mass determination of the collected particulate matter. The overall measurement system is composed of two main parts: the sampling system and the analysis and control system. The sampling head draws in environmental air and, by relying on fluido-mechanical techniques such as direct impaction, virtual impaction, cyclonic filtering, etc., separates the particulate matter fractions. An example of an air sampler is presented in Figure 2. The airflow throughout the sampling system is regulated via suitable control devices that set the flow rates and maintain the operating ranges. The geometry of the sampling head is regulated internationally and is able to select powders with an aerodynamic diameter of less than 10 or 2.5 μm with an efficiency of 50%. A known volume of air is then aspirated for 24 h through the membrane by means of high or low volume sampling systems (i.e., from ≈60 to 1 m^3^/h). The filter membranes have suitable porosity and are made of different materials (quartz, glass fiber, Teflon, cellulose esters, etc.) depending on the type of chemical characterization required for the PM sample. The final concentration of the particulate material is then obtained in the laboratory. After filter conditioning under controlled temperature (20 °C ± 1) and humidity (50 ± 5%), the filter is weighed on an analytical weighing balance, and the change in the mass of the filter divided by the volume of the aspirated gas under reference conditions results in the measurements expressed in $\mu g/m^{3}$.

According to the experience gathered over the recent decades of PM monitoring, PM2.5 measurements may suffer from variability, even when using this standard equipment. This variability can be ascribed to the different sampling system set-ups, but one of the most important problems is the peculiar chemical composition of PM2.5 compared to PM10. The fine-size particle fraction is especially enriched in semivolatile particulate matter (e.g., ammonium nitrate and organic compounds). Hence, problems with losses of semivolatile matter can occur with PM2.5 sampling and could be more pronounced than the losses for PM10 measurements [48]. 

The main drawback of gravimetric methodology is that it provides a measurement of the particulates every 24 h, although it may be important to instead obtain real-time information. Automatic methodologies to enable continuous measurements have been explored, and numerous techniques have been developed and tested, but few techniques have demonstrated equivalence to the reference method described above. The equivalent methods using gravimetric determinations include tapered element oscillating microbalance (TEOM) and beta ray attenuation (BAM, which is not strictly gravimetric but assimilated as such).

For equivalent methods, the sampling system uses the same size-selective PM10 or PM2.5 inlets used for the reference method. Therefore, in the following sections, only the measuring systems will be considered. 

A TEOM makes use of a microbalance system. The tapered element consists of a filter cartridge mounted on the tip of a hollow glass tube. The base of the tube cannot move, while the tip is free to vibrate at its natural frequency (much like the tine of a tuning fork). As particulate matter in the air stream gathers on the filter cartridge, the tube’s natural frequency of oscillation decreases. The mass change detected by the sensor is then the result of measuring the change in frequency: the electronic circuitry senses this change and calculates the particle mass rate from the magnitude of the frequency change. The element is periodically cycled to return it to its natural frequency. To account for humidity or volatile PM components in the sampled air, a filter dynamic measurement system (FDMS) is usually added. This system considers the dynamics of PM deposited on a chilled filter and how that material behaves over time. The flow alternates between base and reference sample periods through switching valves. Since TEOM monitors rely on the frequency of a sensitive oscillating element, mechanical noise may interfere with the calculations. Additionally, dramatic temperature fluctuations can also cause errors due to microbalance. 

A Beta Attenuation Monitor (BAM) uses the principle of β-ray attenuation to measure the mass concentration of PM in ambient air. The air is drawn at 16.7 L per minute (l p m) through a PM10 inlet, followed by using a Very Sharp Cut Cyclone (VSCC) particle size separator to remove particles greater than 2.5 μm for PM2.5 monitoring. The sample stream then passes through a glass fiber filter tape. A carbon-14 (^14^C) element above the filter tape constantly emits β particles, which are detected and counted by a scintillation detector underneath the filter tape. At the beginning of each sampling period, the BAM counts the β-rays attenuated by a fresh, unsampled spot of filter tape. The sample stream is then filtered through that spot of tape. At the end of the sampling period, the BAM counts the β-rays attenuated by the spot of filter tape loaded with the sampled PM. The difference in the degree of attenuation between the pre- and post-sampled filter tape is directly proportional to the mass of PM in the sampled air. The mass concentration in $\mu g/m^{3}$ is then obtained by dividing the PM mass by the total volume of air sampled. 

Beta attenuation analysis also uses an equivalent PM monitor, which combines field-proven sequential sampling technology with BAM measurement principles. The peculiarity of this type of instrument is the presence of two separate inlets and two flow lines with filter holders, which are independently controlled and operated simultaneously. This configuration allows one to sample PM2.5 and PM10 (or PM10-2.5) and yield PM concentration results every hour. 

Another equivalent PM measurement method is the Synchronized Hybrid Ambient Real-Time Particulate Monitor (SHARP), which combines the accuracy of β-ray attenuation monitors with the high time resolution of nephelometers. In this hybrid system, the sample stream first passes through the nephelometer where a sensor measures the light scattering caused by the particulate matter aerosol as it passes through an 880 nm illumination beam. Next, the aerosol is deposited onto a filter tape. Here the instrument measures particulate concentrations by passing radiation through a known sample area to the detector, similar in principle to the BAM, while referencing the β counts according to a reference standard. 

Light scattering is another important technique usually used to measure the particle size distribution of fine particulate material. This technique is used to perform equivalent real-time PM measurements with the precision and accuracy of an FEM instrument. This technique and its underlying principles will be outlined more fully in Section 3 because they form the fundamental principles for LCPMS. 

An accurate list of reference and equivalent instruments for PM measurements can be found in [49]. For SI, Table 3 lists the tools officially recognized by the EPA in 2019.

## 3. Low Cost PM Sensors (LCPMS)

As mentioned in the previous section, the measurement methods analyzed so far require the collection of PM for a period of at least 24 h. As reported in the introduction, over the past few decades, there has been a growing public demand for a more personal, near-real-time monitoring of air quality. As a result, an increasing number of low-cost particulate matter sensor devices suitable for developing IoT tools has appeared. According to the definition introduced by the United States EPA, low-cost PM sensors are characterized by a cost less than USD 1000 and equipped with miniaturized electronics [50]. Due to their low cost, these sensors are poorly documented and not certified. This lack of information has engendered interest in the scientific literature on the capability of such classes of sensors to measure PM. Consequently, several scientific articles have evaluated the properties of such sensors. Table 4 provides an up-to-date list of worldwide LCPMS manufacturers and the number of different PM sensor models they provide, together with the number of scientific papers that have investigated their performance, either in laboratory conditions or in the field. Section 6 reports on the results of this experimental activity to extract a roadmap to better understand the uses and capabilities of these types of devices for the rapid assessment of air quality. 

Notably, all the LCPMS reported above are optical sensors based on an operating mechanism that is different from the sensors analyzed in the previous section. Therefore, to understand the limits and capabilities of this class of devices, we must first clarify how their results can be compared to/refer to the standard measurement systems reviewed above. LCPMS are an evolution/miniaturization of Optical Particle Counters (OPCs) that use light scattering to measure the particle size distribution of materials consisting of fine particles, according to Mie’s theory [51]. Below, the physical operating principles behind all these sensors are discussed, and the OPCs are described. 

### 3.1. Light Scattering: Mie Theory

When a particle passes through a light beam, part of the light deviates from the original path, producing a phenomenon known as scattering; this phenomenon can be exploited to detect the passage of particles provides the foundation of OPCs’ operating mechanisms. 

Scattering is a conservative process resulting from three cumulative effects: reflection, refraction, and diffraction. Several theories proposed during the 1800s tried to explain the dispersion of light but were valid only for particles of certain sizes and shapes and were applicable only in certain media. In 1906, Gustav Mie developed a general theory that rigorously described the light–particle interactions for spherical particles of all sizes dispersed in any medium. The characteristic intensity of scattered light vs. angle variations is precisely predicted by this theory and is called the scattering pattern; by analyzing this feature, the particle size distribution can be determined. The theoretical and experimental constraint assumptions are listed below:

- Light is assumed to be monochromatic and composed of plane waves.
- The particle is spherical and isotropic.
- Both scattering and absorption are considered.
- Light scattered from one particle to another is negligible: this is undoubtedly true if the particle concentration is low.
- The scattering characteristics under consideration are independent of the motion of the particle.
- No quantum effects are considered.

**Figure 3:**
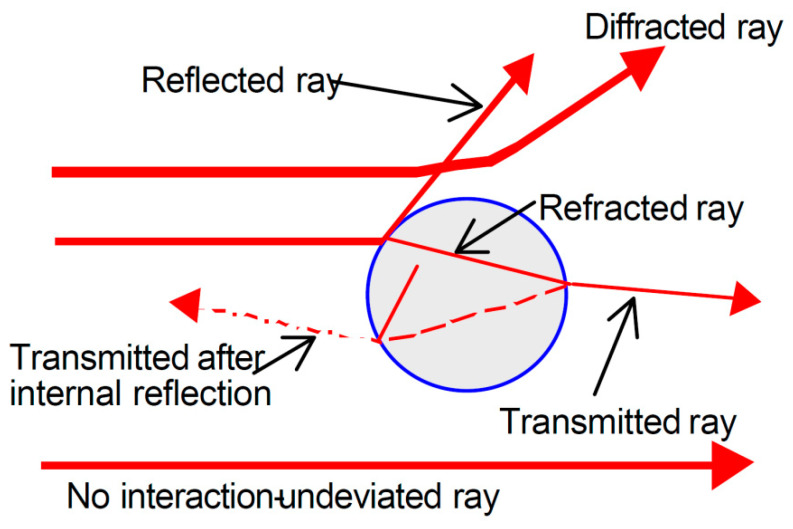
The potential interaction between a light beam and an isolated spherical particle. When the ray and the particle are far enough away, there is no interaction; interaction with the edge of the particle leads to diffraction. When the ray intersects the particle, other phenomena occur, such as refraction, reflection (both internal and external), and transmission. The interaction is considered conservative because there is no absorption (image reprinted with permission of the authors in [52], provided by Micromeritics Instrument Corp.).

In Figure 3, the possible light beam–particle interactions are schematically reported in terms of the relative distance between light beams and particles. More specifically, except for absorption (which is considered a separate phenomenon in the following), the light beam continues its path unaltered if it is far enough from the particle; as the distance decreases, the deviation becomes increasingly more pronounced, passing from a deflection of a few degrees up to an inversion at 180°. 

The intensity of the scattered light is a function of the wavelength λ, the dispersion angle θ (measured with respect to the incident light direction), the particle size *d*, and the relative refractive index *n* of the particle and the medium:

(1) $$I_{sc}=I_{sc}\left(\theta ,\lambda ,d,n\right)$$

Once the angle is fixed and the particle refractive index is known, Mie theory precisely describes scattering. 

Due to the complexity of the relationship between the involved parameters, a simplified form is generally preferred, depending on the size of the particle relative to the wavelength of the incident light [53]. 

Introducing the size parameter α, defined by

(2) $$\alpha =\frac{\pi d}{\lambda }$$

We can distinguish three distinct scattering regimes:

In the case of α∼1, we fall in the Mie Solution regime, and a detailed approach is necessary [54]. The most notable features of these results are the Mie resonances with sizes that scatter particularly strongly or weakly.

If α≪1, we are in the presence of Rayleigh scattering, which describes scattering by particles much smaller than the wavelength of the incident light. In this approximation,

(3) $$I_{sc}=I_{inc}\frac{1+cos^{2}\theta }{2R}{\left(\frac{2\pi }{\lambda }\right)}^{4}{\left(\frac{n^{2}-1}{n^{2}+1}\right)}^{2}{\left(\frac{d}{2}\right)}^{6}$$

where *I_sc_* is the scattered intensity, *I_inc_* is the incident intensity, θ is the scattering angle, and R is the distance between the particle and the light beam. The previous expression returns a dependence on the sixth power of the diameter in this regime. 

Lastly, when α≫1, we are in presence of simplified geometric scattering:

(4) $$I_{sc}=I_{inc}K\left(n,\theta \right)d^{2}$$

Relying on this theoretical background, and to ensure that the intensity of the light scattered is proportional to the particle diameter (0.1–100 µm), optical particle counters are generally designed to operate in the visible or near-infrared range (usually 600–1100 nm).

### 3.2. OPC

**Figure 4:**
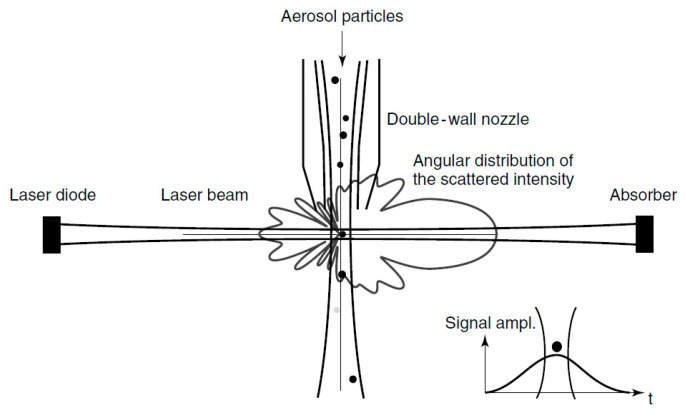
Generic scheme of an OPC: the particle crossing the illumination zone (viewing volume) generates a diffraction pattern at a 360° angle. The corresponding impulse recorded by the photodiode is shown in the inset; the intensity of the signal depends on the particle size, while its width is correlated to the viewing volume (image reprinted with permission of the authors in [59] provided by Wiley—VHC Publisher).

The introduction of OPCs began approximately in the mid-20th century but accelerated in the 1960s after the invention of lasers. The initial goal of particle counting in clean rooms [55,56,57] was subsequently extended to the assessment of air quality and industrial hygiene [58]. This category of instruments uses a light source, typically a focused incandescent lamp or a laser source, to sample a small volume of fluid. It is important to circumvent the simultaneous presence of more than one particle in the same volume to avoid generating errors. Generally, commercial instruments can work with a maximum concentration of 10^6^ particles/liter. For higher concentrations, sample dilution is mandatory, even if this dilution reduces the accuracy of the concentration determination. In practical applications, the particle size distribution is determined by comparing the luminous intensities generated by the passage of each individual particle through a small illuminated area, with a standard curve calibrated using a set of uniform particles with known diameters. To assess the particle concentration, the volume of sampled air is simultaneously measured. Various instrumental configurations are available, thus yielding wide range of instruments; however, the most common configuration involves perpendicular scattering geometry since the perpendicular positioning of the photodetector (θ=90°) limits the effects of stray light inside the device. Briefly, in a laser aerosol spectrometer, a sample of air is drawn into a narrow inlet that limits rogue particles. Then, a collimator generates a laser curtain perpendicular to the inlet stream. When the infrared laser light illuminates particles in the air sample stream, a scattering signal is produced and detected at an angle of 90° by a photo diode (see Figure 4). In accordance with Mie theory, each measured pulse height is correlated to the particle size.

Part of the scattered light at a certain acceptance angle is collected by a photodiode. The amplitude of the detected signal is then compared to the standard calibration curve obtained from the known particle sample. 

**Figure 5:**
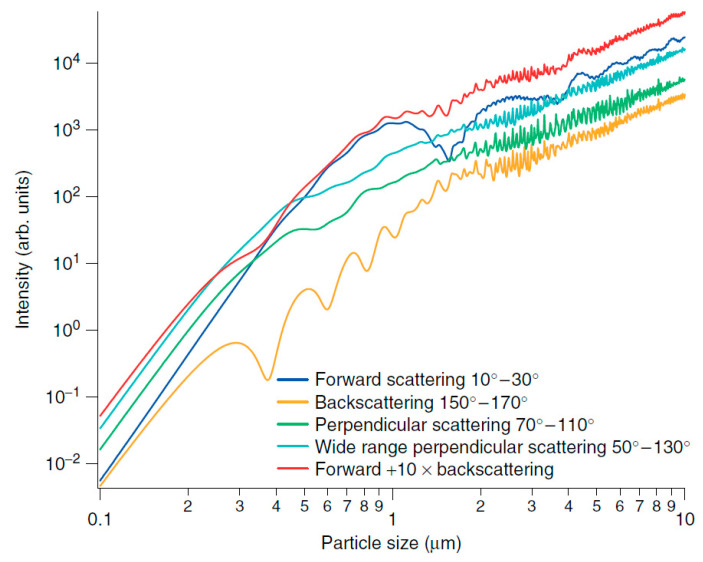
Calculated calibration curves for different scattering angles and integration ranges for polystyrene latex: in the case of forward and back-scattering, the relationship between size and scattering intensity in some size intervals, namely the 0.4–1 μm range for back-scattering and the 1–3 μm range for forward scattering, is not uniform. In the case of perpendicular scattering, shorter oscillations are observed in the range of 2–5 μm; this latter geometry is preferable both for its dimensional distribution and because it has less dependence on the refractive index of the particles (image reprinted with permission of [59] provided by Wiley—VHC Publisher).

As an example, the calibration curves calculated by Aladar Czitrovszky [59] using Mie theory for different scattering and integration angles are shown in Figure 5. From these curves, the relationship between the size and the scattered intensity in certain size ranges is shown to be nonuniform. For back scattering, long-period oscillations are prominent in the sub-micrometric range, whereas for forward scattering, these oscillations are pronounced in the 1–3 µm range. Once again, perpendicular scattering is confirmed to be the best geometric configuration, with short period and small intensity oscillations confined in the 2–5 μm range. 

The output of an OPC is a signal monitored over time, composed of a sequence of pulses; the intensity of each pulse represents the light scattered by a particle passing through the OPC and is related to its dimension through a calibration curve, while the FWHM of the signal is associated with the volume of the instrument viewing region [60,61]. The particles are counted and tabulated into standardized counting bins. In this way, by considering the air flowing through the OPC, the concentration of particles in the air and their dimensions can be determined.

## 4. LCPMS Characterization and Calibration

In recent years, different scientific works have contributed to evaluating the operation of several LCPMS devices. The increasing numbers of these types of sensors and the various evaluation techniques proposed for their parameters raised doubts about how to evaluate and define their performance. Only two years ago, in 2018, the EPA finally defined the characteristic parameters for this class of devices (see SI). Since then, several papers have reported on LCPMS calibration and characterization using different types of setups, including laboratory conditions, partially controlled conditions, and field deployment conditions. In the following section, a review of several of these relevant works is provided. Section 4.1 focuses on laboratory and partially controlled setup characterizations, while Section 4.2 addresses field characterizations. Finally, in Section 4.3, publications about calibration techniques and their results are specifically reviewed. 

### 4.1. Laboratory Characterization

The deployment of affordable PM sensors requires a great deal of effort to assure their data reliability. These sensors must be calibrated using reference instruments and fully characterized under different environmental conditions. Accordingly, the development of characterization chambers in the laboratory is crucial to facilitate realistic testing in a controlled environment. The characterization phase in the laboratory, under controlled environmental conditions and pollution levels, allows the performance assessment of low cost sensors before their field deployment and the evaluation of possible differences in their responses within the same family of LCPMS, the latter being of particular relevance to their application in sensor networks [62]. 

**Figure 6:**
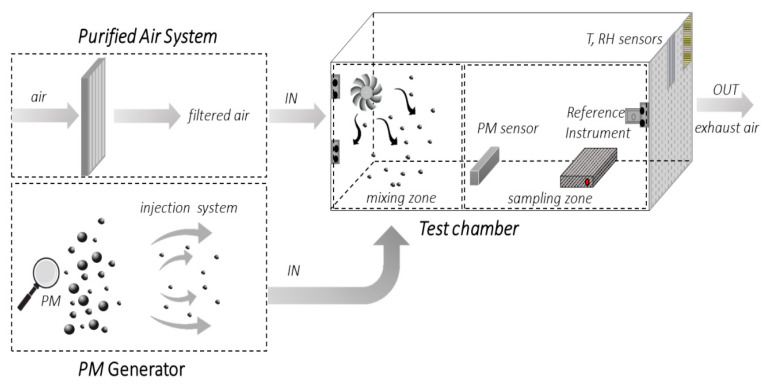
A schematic of the PM characterization system: PM generator, purified air system, and test chamber with PM sensors, Reference Instruments and T, RH sensors.

In general, an aerosol chamber consists of three principal sections: a test chamber (TC), which is isolated from the external environment and equipped with a PM reference instrument and humidity (RH) and temperature controls; a purified air system, which is necessary to provide clean reference air in the TC; a PM generator that ensure a controllable PM concentration inside the test chamber. A general schematic of an aerosol chamber for PM measurement is depicted in Figure 6.

**Figure 7:**
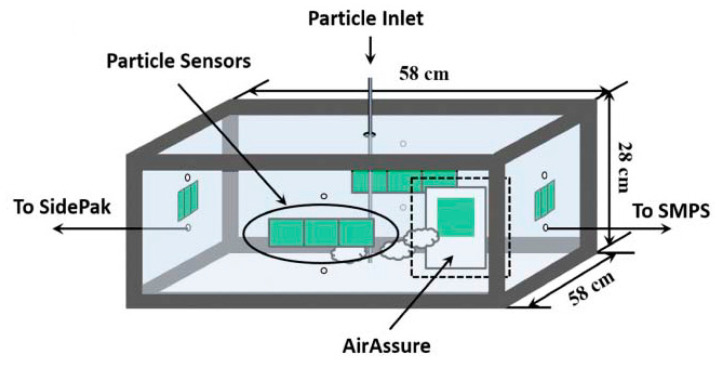
Schematic of the chamber for particle measurements and the arrangement of particle sensors, developed by Wang Yang (2015). A SidePak Personal Aerosol Monitor AM510 (TSI Inc.), a scanning mobility particle sizer (SMPS, TSI Inc.), and an Air-Assure PM2.5 Indoor Air Quality Monitor (TSI Inc.) were used to provide reference measurement results to evaluate the performance of the sensors (image reprinted with permission provided by Taylor and Francis and Copyright Clearance Center—License Number 4836480810613).

Over the last few years, several types of test chambers have been proposed. One of the earliest test chambers was realized by Yang Wang in 2015 [63]. As shown in Figure 7, a custom-built acrylic glass chamber was assembled (58 × 58 × 28 cm, chamber volume of 94.19 L) with its edges sealed using rubber strips to avoid PM losses. 

The authors positioned three LCPMS (Samyoung DSM501A, Shinyei PPD42NS, Sharp GP2Y1010AU0F) onto each vertical wall to minimize the spatial differences in PM concentration. Particles were produced from NaCl, sucrose (C_12_H_22_O_11_), and NH_4_NO_3_ aqueous solutions, and atomized by an aerosol generator (Model 3076, TSI Inc.) and the burning of incense (Sandalum Agarbathi Cones, Cycle Brand). The particles were dried, sent to the test chamber, and suspended through fans. The apparatus was equipped with a sensor probe and a thermocouple for controlling the temperature and humidity during the test. A SidePak-TSI Inc. (St. Paul, MN, USA), a scanning mobility particle sizer (SMPS-TSI Inc.), and an AirAssureTM PM2.5 (TSI Inc.) were used as reference instruments and located outside the test chamber. All three LCPM sensors showed high linearity against SidePak with incense particles up to a 0–300 µg/m^3^ concentration range. In particular, the authors obtained a correlation coefficient *R*^2^ of 0.9525 for Shinyei PPD42NS, 0.9755 for Samyoung DSM501A, and 0.9746 for Sharp GP2Y1010AU0F. Moreover, this work showed how the humidity and particle size/composition affected the sensor performance, which did not happen with temperature. 

**Figure 8:**
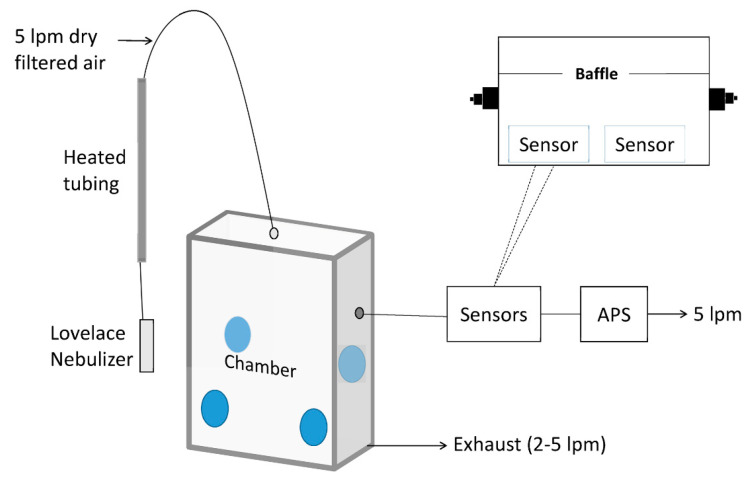
Schematic of the experimental set-up realized by Austin’s group. The blue circles indicate the location of the mixing fans inside the chamber (2015) (image reprinted under the terms of the Creative Commons Attribution License which permits unrestricted use; PLoS ONE 10(9), e0137789, doi:10.1371/journal.pone.0137789).

That same year, Austin et al. built a small airtight box (6 × 21 × 8 cm, Total Volume 1 L), which was further reduced by placing a fixed baffle along the box, as reported in Figure 8 [64]. Four LCPM sensors (Shinyei PPD42NS) were placed in this box. Monodisperse polystyrene spheres and polydisperse dust (ASHRAE test dust #1) were used as the particulate sources and were nebulized before entering the chamber. Dry filtered air was used as the baseline for the measurements. Particulate aerosol was injected using steel tubes into the first chamber, where the particulates were suspended by four mixing fans. Then, the particulates were sucked towards the sensors using the internal pump of the TSI APS (Aerodynamic Particle Sizer), the reference instrument, in series with the Shinyei sensor chamber. Aerosol was generated until the particulate concentration achieved a specific value. Then, the aerosol injection ceased along with the decay of the particles measured using both the APS and the sensors in series. This comparison demonstrated that the precision of the Shinyei sensors compared to the APS can reach as high as 0.99 *R*^2^ for monodisperse aerosols in a concentration range of 0–50 μg/m^3^. The authors concluded that the Shinyei PPD42NS sensors have appropriate sensitivity for mono disperse aerosol, but in cases of polydisperse aerosols with unknown composition, the conversion to mass was possible only using a gravimetric method.

**Figure 9:**
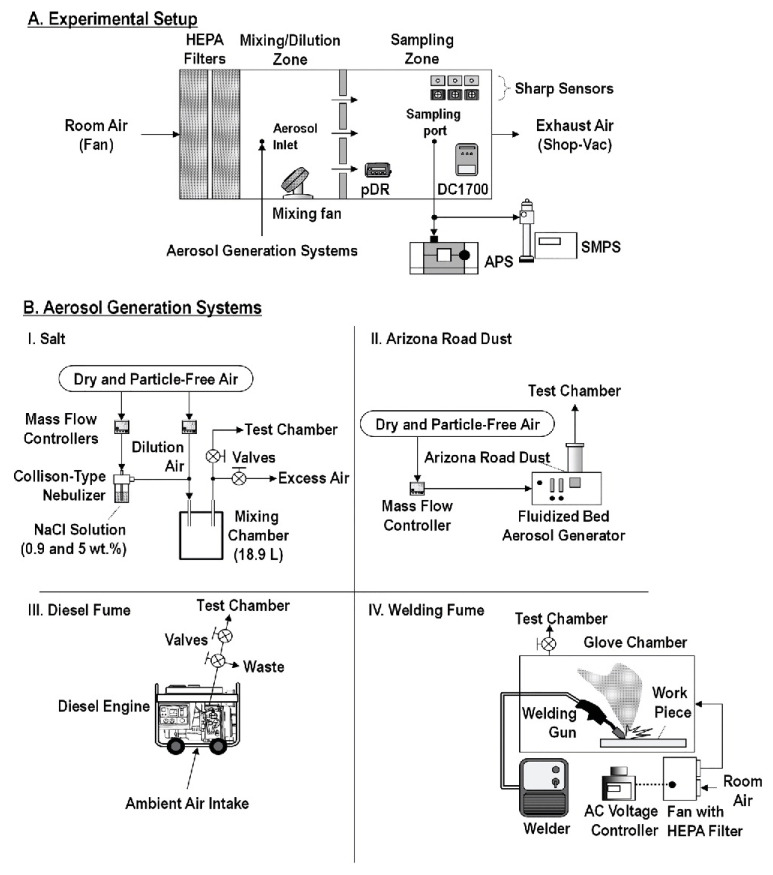
Experimental set up used by Peter’s group (2016) (**A**). Schematics of the aerosol generation systems shown in panel (**B**) (image reprinted with permission provided by the Taylor and Francis and Copyright Clearance Center—License Number 4836480661138).

The next year, Thomas Peters’ group [65] developed a more complex system to evaluate low-cost sensor performance under high particle concentrations related to occupational applications (up to 6500 µg/m^3^). In the experimental setup depicted in Figure 9a, a single chamber was divided by a perforated plate into two distinct zones: a mixing zone (64 × 64 × 66 cm^3^) and a sampling zone (53 × 64 × 66 cm^3^, V 224 L). The perforated plate (600 holes with a diameter of 0.6 cm)) ensured a uniform distribution of the particles. Zero air (air cleaned through HEPA filters) diluted the generated aerosol. Particles were put under laminar flow (0.01m/s), and three DC1700 sensors, two Sharp sensors (Sharp GP and Sharp DN), and one pDR-1500 photometer were placed in the sampling zone. The laboratory analytical instruments located outside the chamber included a condensation particle counter (CPC; 3007, TSI Inc., particle diameter ≤0.3 µm) and an aerodynamic particle sizer (APS; 3321, TSI Inc., particle diameter ≥1 µm). In addition, a sequential mobility particle sizer (SMPS-C 5.402, GRIMM) ensured that the particle generation was monodispersed. The authors tested the four particle types (Arizona dust, salt, diesel exhaust, and welding fumes), which were generated in different ways (Figure 9B). In detail, the salt droplets were obtained through a Carefusion nebulizer, a fluidized bed generator (3400A, TSI Inc.) produced an aerosol of Arizona dust, diesel waste fumes were derived from a diesel generator, and welding fumes were extracted from a welding apparatus. The LCPM sensors compared to the photometer (pDR-1500) showed a high correlation coefficient (R^2^ > 0.97) and high precision (<8%).

**Figure 10:**
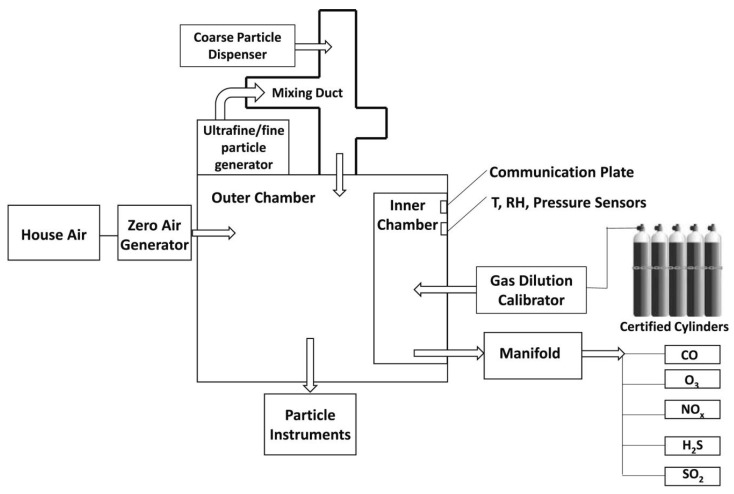
Schematic of the chamber system developed by Papapostolu et al. (2017) (image reprinted with ELSEVIER PERMISSION—License Number 4833500101157).

Papapostolou et al. developed the test chamber represented in Figure 10 [56]. This chamber demonstrates the possibility to introduce several gases at the same time; this chamber also controls the temperature, pressure, and relative humidity and uses a constant particle concentration. Dry, particle-free systems are used to generate the reference air. Specifically, the equipment consists of two chambers: the outer chamber and the inner chamber. The former is a stainless steel rectangular (volume around 1300 L) measurement box featuring LCPM sensors and reference instruments (GRIMM (EC180-FEM), TSI (Model 3321-BAT), and TSI (Model 3091-BAT)). The inner chamber is a Teflon-coated stainless-steel cylinder (volume 110 L) used for gas sensor monitoring. This configuration allows the direct comparison between the LCPM sensors and the reference instruments during the measurement. Two different aerosol generators were introduced to generate ultrafine/fine particles (model AGK 2000, PALAS, Karlsruhe, Germany) and fine/coarse particles (model SAG 410/U, TOPAS, Dresden, Germany). The authors conducted experiments to demonstrate the system’s ability to realize stable and reproducible aerosol and gaseous atmospheres under a wide range of temperature (T) and relative humidity (RH) conditions. 

This chamber is used by the well-known South Coast AQMD (Air Quality Management District) research center, which set up the programme AQSPEC (Air Quality Sensor Performance Evaluation Center) [66]. The main aim of this programme is to inform the general public about the effective performance of low-cost environmental sensors, that can be easily found on the market, by clarifying their actual performance both in the laboratory and on field (see next paragraph for the related protocol). Moreover, the programme aims to highlight the advantages and possible limitations that low cost sensors could have. As already said, the chamber gave results for the LCPMS, in which 3 units of the same sensor are inserted into the chamber to obtain as much statistical information as possible, in particular on the degree of correlation with the reference instruments and their reproducibility.

**Figure 11:**
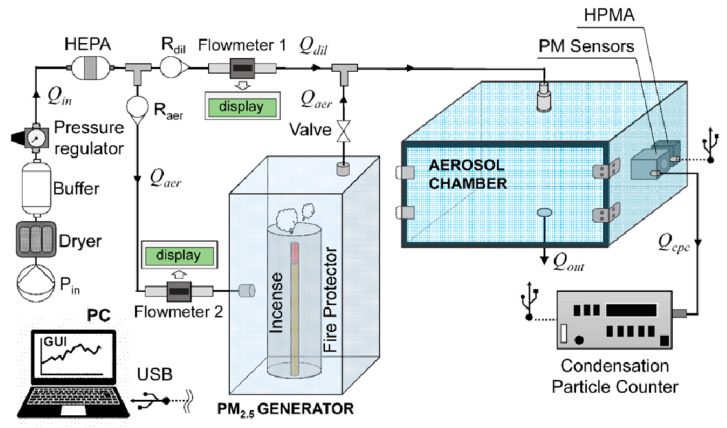
Experimental setup to characterize the aerosol chamber and evaluate the PM sensors of Hapidin (2019) (image reprinted under the terms of the Taiwan Association for Aerosol Research and Aerosol and Air Quality Research. All articles published on this site use the Creative Commons Attribution 4.0 License (CC BY 4.0); Aerosol and Air Quality Research, 19: 181–194, 2019; doi:10.4209/aaqr.2017.12.0611).

Subsequently, Hapidin’s group achieved the experimental setup reported in Figure 11 [67]. The authors adopted an exponentially decaying particle concentration to evaluate three commercial PM sensors (Sharp GP2Y1010AU0F, Winsen ZH03A, and Novafitness SDS011), as already seen in Wang’s work [63]. The authors noted that, compared to other test methods that use constant particle concentrations, this concentration condition can considerably reduce the testing time [56,64,65], as this apparatus is equipped with further output airflow to accelerate the PM concentration decay. The aerosol chamber was custom-built in acrylic material (with a volume of 70.53 L). In the test chamber, two reference instruments (a CPC-TSI, model 3025A and a factory-calibrated particle sensor, Honeywell, model HPMA115S0-XXX) were located next to three LCPM sensors (Sharp GP2Y1010AU0F, Winsen ZH03A, and Novafitness SDS011). The aerosol was generated by burning incense and sent to the test chamber until reaching a $1000\mu g/m^{3}$ concentration (the HPMA detection limit). In this system, a pump with silica gel and HEPA filters ensured a dry and clean input airflow. The results showed good linearity towards the HPMA reference monitor, with *R*^2^ values of 0.999 for the Novafitness SDS011 at a low particle concentration ($0\text{–}400\mu g/m^{3}$). In addition, the Winsen ZH03A sensor and Novafitness SDS011 sensor presented coefficients of variation below 10%, which is within the precision envelope set by the EPA standards.

**Figure 12:**
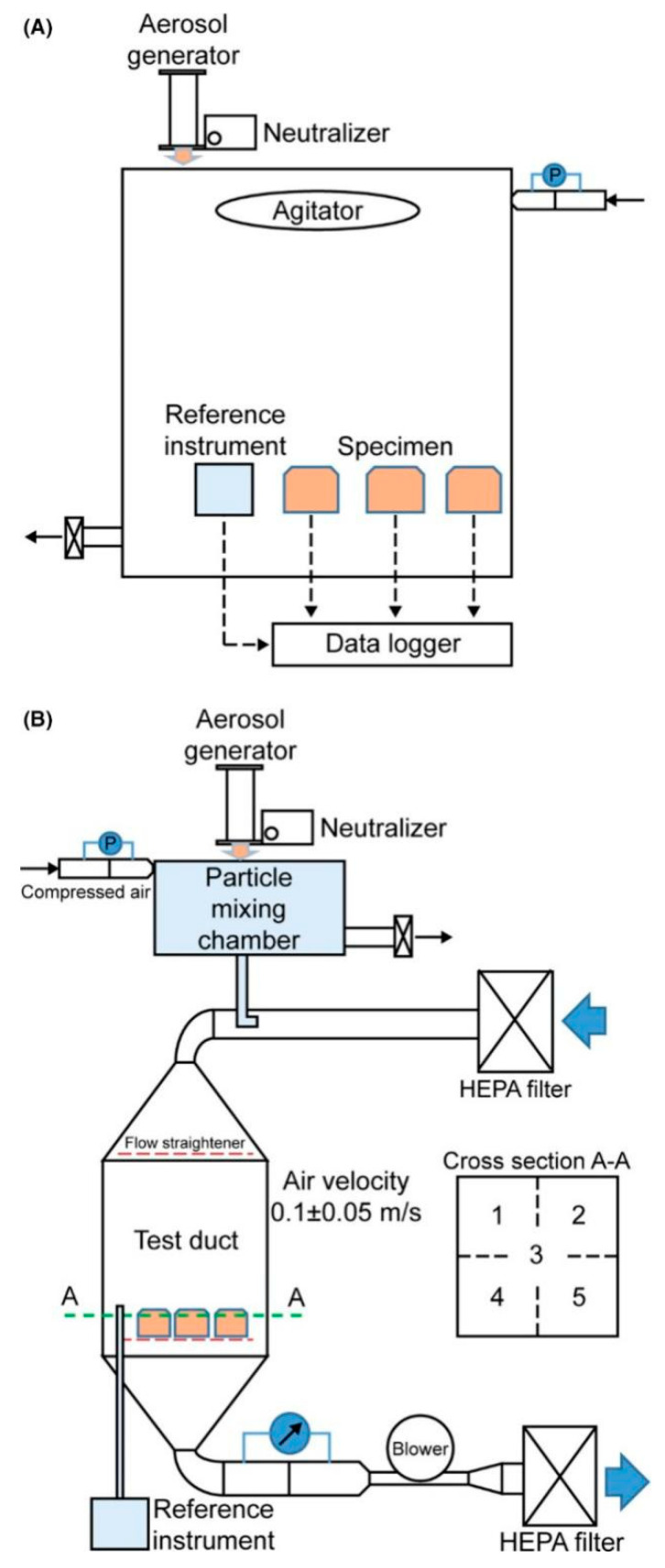
Schematics of low-cost dust sensor evaluation systems: (**A**) chamber test; (**B**) low-speed duct test (image reprinted under the terms of the Creative Commons Attribution 4.0 License (CC BY 4.0); Indoor air, 30(1), 137–146, doi:10.1111/ina.12615).

In the work by Kim et al. [68], two types of test systems (Figure 12) were employed. One used a mixing chamber (50 L), where particles were mixed with clean air, and the overall airflow velocity was carefully adjusted to ensure that the dust sensor would function properly (Figure 12A). In the second test system, an exponentially decaying particle concentration was realized in a low air-speed duct (Figure 12B). Particles generated by an atomizer were first introduced to a particle mixing chamber until the particle concentration reached its proper level. Then, the particle inlet was closed, and clean air was supplied to the particle mixing chamber. A 5 wt% potassium chloride (KCl) solution was used to generate particles through an atomizer for both test systems. The reference instrument for measuring the size distributions was a Grimm 1.209 dust monitor (Model 1.209, Grimm Aerosol Technik Company, Ainring, Germany). Large-scale comparison work was then carried out. The authors tested about 264 LCPM sensors and classified them into four different groups (Classes 1, 2, 3, and 4), depending on their performance with respect to the reference instrument. Most of the sensors showed very good linearity (slope of concentration measurement data) with the reference data. About one quarter of the tested sensors satisfied the Class 1 acceptance limit. However, accurate PM10 measurements were rarely achieved, likely because of the difficulty in transporting large particles to the detection zones. 

**Figure 13:**
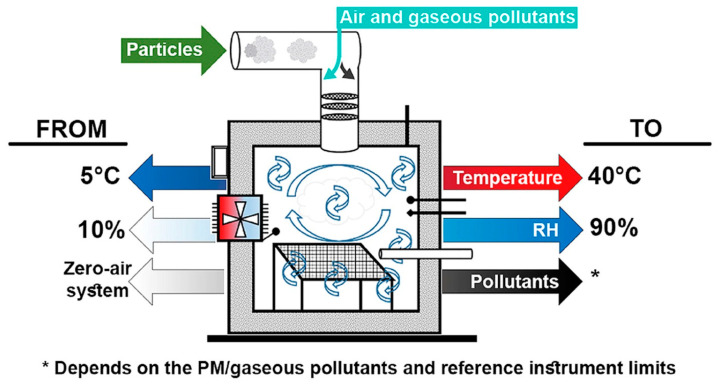
Schematic of the chamber system with the main components developed by Omidvarborna et al. (2020) (image reprinted with ELSEVIER PERMISSION—License Number 4834170480943).

Following Papapostolu’s measuring method, Omidvarborna et al. designed an environmental-pollution chamber to test LCPMS performance under a controlled temperature and relative humidity (RH). Different particle types and pollution gas concentrations could be generated in this chamber [69]. The authors constructed the test chamber (50 × 50 × 50 cm^3^, V 125 L) from acrylic sheets sprayed with Teflon isolated by 100 mm of Styrofoam (Figure 13). The sheets were used to minimize the surface reactions for the gaseous and aerosol experiments. The interior edges of the chamber were sealed with rubber strips to prevent leakage. The environmental chamber was connected to a humidifier/dehumidifier system, a heat pump, a particulate matter (PM) generator, a gaseous air pollutant supply (ozone ($O_{3}$), NO, ${\mathrm{NO}}_{2}$, ${\mathrm{SO}}_{2}$, hydrogen sulphide ($H_{2}S$)), and reference measuring instruments (a Vaisala HMT120 temperature/RH sensor and an optical particle counter, Grimm EDM 107). This chamber was able to reproduce both outdoor and indoor environmental conditions (a temperature range from 5 to 40 °C and an RH from 10% to 90%) and stable pollutant concentrations. After comparing the results obtained with the temperature/RH sensors and the HPMA115S0 Honeywell particulate sensor to those obtained by the reference equipment, the authors found good correlation, with *R*^2^ values above 0.96, 0.99, and 0.97 for temperature, RH, and PM2.5, respectively. The results, according to the authors, showed that this chamber could be particularly interesting for its affordable cost, small size, light weight, and ease of operation. This chamber generated similar environments and pollutant concentrations to evaluate the performance of LCPMS under various conditions. 

**Figure 14:**
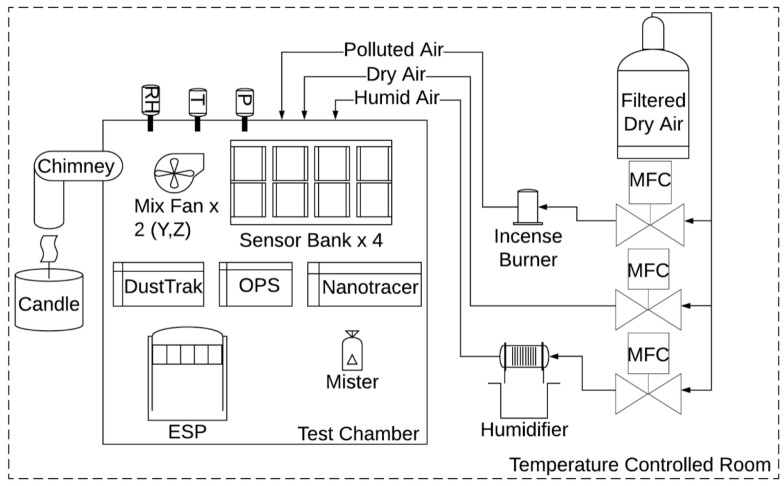
Schematic showing the arrangement of the test chamber and the supporting equipment (image reprinted under the terms and conditions of the Creative Commons Attribution (CC BY 4.0) license—License MDPI, Basel, Switzerland Sensors 2020, 20, 2219; doi:10.3390/s20082219).

In one of the most recent works, Cox et al. applied an exponentially decaying particle concentration with PM2.5 median peak concentrations <40 μg/m^3^ using two different pollution sources to test 40 LCPM sensors (produced by five manufacturers) [70]. The PM from incense stick burning, was sent to the chamber through dry, filtered air (5 L/min flow) using 1179A MFC (Figure 14), while the PM generated by candle burning was sent into the chamber wall via an angled chimney. This chimney was kept sealed for all the remaining time. The authors used a 1 m^3^ Perspex/stainless steel chamber framed by aluminum profiles. Fans ensured air mixing within the chamber. A walk-in, temperature controlled room (Viessmann A/S; setpoint within 25.9 and 28.7 °C) contained the chamber, while the RH was varied between 55% and 90% within the chamber through the modulated introduction of filtered air, dry air, or humidification via a Nafion membrane MFCs (MKS type 1179A 20 L/min plus type 1579a 100 L/min). The chamber pressure was left to reach equilibrium prior to start each test. The authors characterized eight different LCPMS models for each of the five different manufacturers: Honeywell HPMA115S0, Alphasense OPC-R1, Novafitness SDS018, Sensirion SPS030, and Plantower PMS5003. Eventually, the sensor performance was evaluated through the reference measurements obtained by a DustTrak DRX 8533 Desktop device (TSI Inc., Shoreview, MN, USA), an Optical Particle Sizer OPS 3330 (TSI Inc.), and an Aerasense Nanotracer (Oxility BV, Best, Netherlands). Using this test system, the authors highlighted the different behaviors of the tested LCPMS under different PM types and relative coefficients of variation based on their responses to peaks or stable concentrations of particles. The results allowed the authors to compare the performance of different LCPMS models. The coefficient of variation reached higher values when comparing responses to peak values rather than comparing the stable concentrations of particulates for all sensors. Alphasense OPC-R1 produced the highest coefficients of variation under both peak and stable conditions. However, all sensors showed better performance when dealing with candle-generated PM. In particular, the Sensirion SPS030 and Plantower PMS5003 obtained similar scores regardless of the rate of concentration change (either peak or stable concentrations). The remaining three sensor models presented lower scores for stable concentrations, indicating better performance under peak concentration regimes. The authors concluded that the Sensirion SPS030 obtained the lowest coefficients of variation among all models of sensors, irrespective of the testing scenario.

In all the above-mentioned works, the production of particulates and their injection into a test chamber are key issues. To create a uniform environment in terms of concentration and composition, controlled systems are generally used. Indeed, some authors use completely automated aerosol generators to obtain stable and reproducible particulate environments [56,64]. In one case, an automated aerosol generator (TOPAS SAG 410) was coupled with an aerosol wind tunnel [71]. In this study, the particulate was maintained in a flow at low-wind speeds (below 0.5 m/s) to reproduce values typical of most occupational environments. The wind tunnel was 1.22 × 1.22 × 6 m^3^, with the sampling zone possessing a length of approximately 3 m and the airflow through the wind tunnel was generated by four fans. This work showed that the fan speed directly influenced the particle concentrations: the higher the fan speed was, the higher the chance of a particle hitting the chamber walls, leading to lower aerosol concentrations.

Other authors proposed homemade systems to obtain PM from more easily accessible materials. In Ristovski’s lab, cigarette and e-cigarette smoke was studied as an indoor particulate source [72,73]. In other works, various indoor deodorants, such as aroma diffusers, Armenian paper, incense, candles, and instant or automatic sprays, were used [74]. Incense burning is a particularly useful particulate source since it produces a monodispersed aerosol suitable to represent PM2.5 [63,67,75,76].

**Figure 15:**
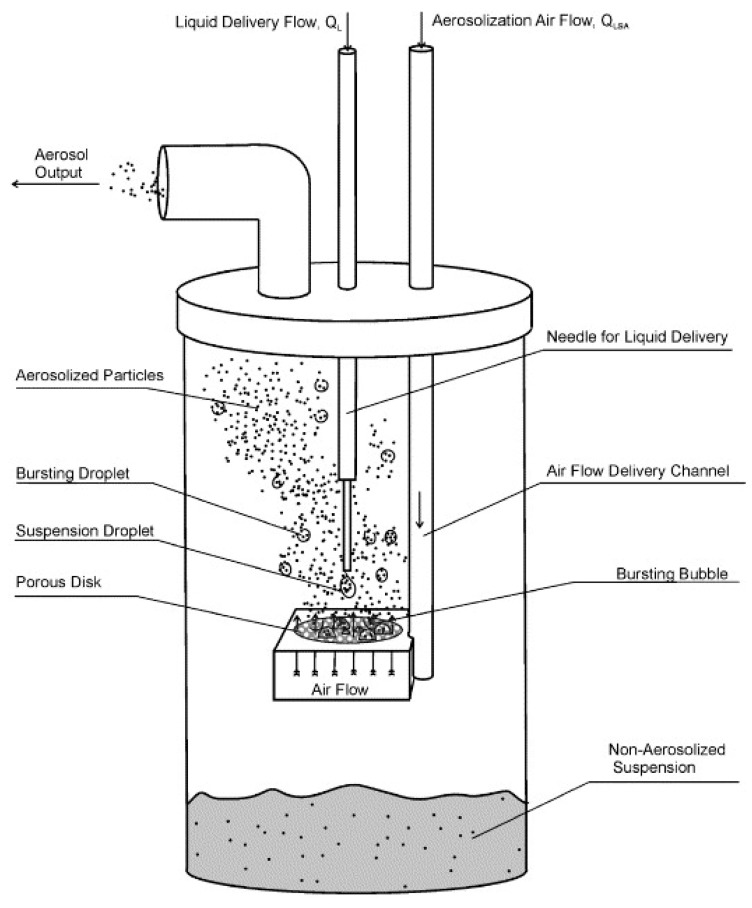
Schematic diagram of collision nebulizer (reprinted with ELSEVIER PERMISSION—License Number 4834190009888).

In addition to particulate production, several methods were proposed to introduce the PM to the chamber test. Zamora’s group used three different methods according to the PM source. For incense, a stick was lit and situated inside a holder until the required concentration was obtained. Then, the stick was removed, and the decrease in the concentration was observed; talcum powder was also investigated after being dispersed with filtered air and injected into the test chamber. Sodium chloride (NaCl) and oleic acid were also used in a collision nebulizer (CH Technologies, Westwood, NJ, USA) (shown in Figure 15). In this case, the filtered air flowed through the nebulizer, which was filled with either a NaCl water solution or pure oleic acid until reaching the desired concentration of NaCl or oleic acid; then, the input air flow was turned off [77].

**Figure 16:**
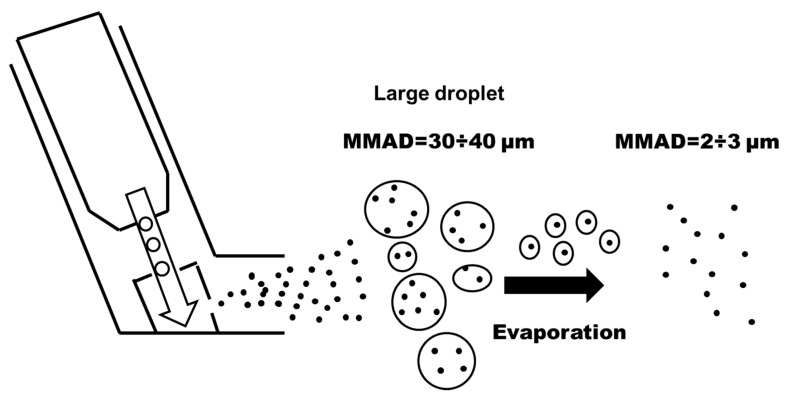
Diagram of a typical pressurized metered dose inhaler showing the mechanism of particle formation (MMAD = mass median aerodynamic diameter). Adapted from Figure 1 in [78].

Some authors also proposed to use commercial pressurized metered dose inhalers (pMDIs), such as Atrovent and Ventolin. By pressing the bottom of the canisters of such inhalers, a well-defined drug dose is released. The canister contains the drug in a powdered form dispersed in a liquid propellant with other additives [78]. The authors evaluated the particle dimension by means of the MMAD (Mass Median Aerodynamic Diameter): when the bottom is pressed, a mixture of the drug and aerosol is formed, whose particle diameter depends on the propellant evaporation and the distance from the outlet. At the outlet, the particle diameter is 30–40 µm including the aerosol cloud surrounding the particles, while at a distance of approximately 10 cm from the valve, the diameter decreases to 2–3 µm (Figure 16).

To use this system for particle production, several boundary conditions need to be assessed in the overall context of the experiment. Rui Zhang investigated the effect of environmental conditions (temperature and humidity), flow rate, and device temperature on the size the measurement of particles emitted through pMDI [79].

### 4.2. Field Characterization

LCPMS field characterization is a fundamental operation, even when preceded by laboratory characterization. Even with the best technologies available, one of the main problems is replicating real conditions since the diversity of the chemical–physical compositions of particulates can only be definitively evaluated in the field.

For field characterization, after performing tests in the laboratory as mentioned above, the authors in [77] tested their sensors in an indoor environment (an apartment of $1000\;{\mathrm{ft}}^{2}$; in particular, the campaign monitored a kitchen) using a pDR-1200 (TEOM) as a reference. For the outdoor measurements, the LCPMS network was placed at the University of Baltimore MDE at an important intersection at a height of 5 m; in this location, about 50,000 vehicles pass every day. The measurements were obtained in the summers of 2016 and 2017 and applied both a gravimetric system and a BAM (FEM), reaching a linear correlation coefficient $R^{2}$ of 0.92 with the PM reference measurements.

In [80], Plantower sensors were tested at an important road junction near Salt Lake City. The sensors used a sampling frequency of 1 min and, depending on whether they were compared to an FEM or FRM, the data were averaged over 1 or 24 h. The results showed that the linear correlation coefficients with the FRM vary widely depending on the seasons, reaching their minimum in spring (0.185 for PMS1003 and 0.419 for PMS5003), while the maximum correlation was observed in winter, at which time the $R^{2}$ rose to 0.972 and 0.971, respectively. Moreover, the authors indicated a significant drift after 3 months when using the pms1003.

The authors in [81] developed a one-year comparison between four different LCPMS (Alphasense OPC-N2, Plantower 5003, Plantower 7003, and Honeywell HPMA115S0), which were all placed near two schools in Southampton, with respect to a reference British station (AURN). The nearest AURN PM2.5 monitoring station was located in Portsmouth, 40 km to the East, and was equipped with FDMS 8500 and TEOM 1400ab Ambient Particulate Monitors, which reported the hourly PM2.5 concentrations along with the volatile and nonvolatile PM2.5. The comparison shows that the two Plantower sensors exhibited coefficients of linear correlation ($R^{2}$) always greater than 0.8, while for OPC-N2, the $R^{2}$ was always less than 0.7.

In [82], a six-month measurement campaign was performed with four LCPMS (SDS011, ZH03A, PMS7003, and OPC-N2) at the Meteorological Observatory of the Department of Climatology and Atmosphere Protection of the University of Wrocław. The reference systems provided by this site were TEOMs. In this work, the Plantower again showed the best linear correlation with respect to the reference instrument, reaching an $R^{2}$ of 0.93 when its raw data were averaged over 24 h. The authors analyzed how the correlation coefficient varies according to the measurement range for the Novasense and Plantower by dividing the latter into three zones: (1) $20\;\mu g/m^{3}$, (2) $20\text{–}60\;\mu g/m^{3}$, and (3) $60\;\mu g/m^{3}$. Surprisingly, at very low concentrations and in the first zone, no sensors were able to reach a linear correlation coefficient of 0.6 (both with 15 min and 1 h averages).

In [83], Alvarado et al. sought to characterize a low-cost dust sensor (Sharp GP2Y10) in an unusual way. The authors installed two sensors on two different UAVs (on the fixed and rotary wings), where the air suction and exhaust were modified to obtain controlled flow inside the sensor. Bench tests were conducted using incense burning, and nonlinear regression was used to calibrate and assess the potential correlation between the sensors and a commercial industrial grade analyzer (DustTrack 8520) at very high concentrations (0–120 $\mathrm{mg}/m^{3}$), resulting in relative errors ranging from 12% to 22%. After a careful analysis to filter the noise produced by switching the electric motors of the UAV, the authors performed controlled operative condition tests to reconstruct the 3D particulate concentration fields. One of these tests included pumping talcum powder with a garden blower, so the sensors were recalibrated to handle the composition of these new particulates. The authors concluded that the integration of air quality sensors and autopilot data is potentially feasible and would allow one to determine the distribution of airborne particulates in time and space. However, the authors advocated for using novel optical sensors with higher sensitivity and calibrations that can exclude cross contamination for more realistic concentration scenarios.

As stated in the previous paragraph, AQ-SPEC provides evaluations for LCPMS operating on field, in addition to the ones they provide for laboratory setup. In the former, the low-cost sensors are compared with FRM or FEM monitoring instruments, usually used for the detection of pollutants for regulatory and legislative purposes. In particular, for PM detection, the research center uses a GRIMM EDM180, which is a PM meter based on an optical system (like most of the LCPMS); it is considered as a FEM by EPA, and provides for real time particulate concentration measurements. In addition, they also rely on two MET ONE 1020 (one for the PM2.5 and one for the PM10), which are beta ray attenuators providing hourly averages of the concentration of particles in the air [84]. For on field evaluations, the AQ-SPEC researchers refer to a strict protocol, in which, after verifying the correct match between the ‘bench’ test and the documentation (power supply specifications, evaluation of the data acquisition method), the LCPMS are inserted in the monitoring station for 30-60 days. The sensors are checked to be functioning and recording with a weekly basis [85]. The tests, reported in [86], show (1) sensor picture, (2) producer and model of the sensor, (3) price, (4) tested pollutant (PM2.5, PM10, etc.), (5) coefficient of determination *R*^2^ on the field, (6) coefficient of determination *R*^2^ in laboratory, and (7) a summary report which provides the link of each specific sensor. Their efforts, along with those of other organization such as EPA [87], for building a crystal-clear picture of the effective performances of PM monitors and LCPMS both on field and in laboratory, using top category reference instruments, is paramount for the general public to understand real world capabilities of these tools and for practitioners while challenged by the choice of (low-cost) instruments for the environmental control. More important, their work is supportive for other researchers regarding the realizations of test chambers for environmental sensors and the development of a measurement protocol for evaluations on field.

All the papers indicate fundamental differences between laboratory tests and field tests. In a field characterization, infrastructure that can record data for several months with the instruments left unattended for several days or weeks is necessary. The results of these long-term characterizations depend on their correlation with a reference instrument, but the accuracy is difficult to estimate since it depends on the relative position between the LCPMS and the reference instrument.

### 4.3. Calibration of a Low-Cost PMS

Calibration of the LCPMS is a crucial and mandatory operation. Indeed, all LCPMS do not directly measure size-partitioned mass concentrations but rely, instead, on basic a priori assumptions of particle size and distribution to derive their estimations. Whenever these initial assumptions are not met (for example, due to exposure to a different particulate composition), the LCPMS are expected to express low accuracy, low precision, and nonlinearity effects when estimating particle concentrations. However, the validity of these assumptions depends on the particular field conditions of the operative environment, which can constrain their usage. As a further example, operational upper measurement level limits in the range of 5 to 10 hundreds of $\mu g/m^{3}$ are usually required, which practically rules out several cities that often report higher PMx concentrations [88]. It is also important to note that LCPMS are usually provided with very limited information, if any, regarding their factory calibration schemes.

LCPMSs rely on the particle density hypothesis to translate the particle size and count measurements in mass concentration estimations. As noted, the estimation of size-partitioned particle concentrations involves several physical and chemical factors that may affect the accuracy and precision of the data. Moreover, these effects strongly depend on the characteristics of the LCPMS and specifically on the peculiarities of the general sensing mechanism itself. Among the different factors, Lewis et al. [89] focused on chemical composition and reactivity, refractive index, temperature/humidity gradients along the sampling path, and shape and size distribution. Particle size distribution may also significantly affect accuracy due to size detection limits. This may severely hamper the particle count of small (<500 nm) particles (see also Figure 1), thereby underestimating the mass concentration in the lowest partition bin (e.g., PM2.5 or PM1), which is usually related to ultrafine particle measurements that are increasingly relevant in urban environments, where a significant fraction of particle pollution is derived from car traffic. Furthermore, size distribution may be affected by the actual sampling setup, such as the use of long or bended manifolds.

The application of an ad-hoc calibration function is as follows:

(5) conc=f(s,i)

where s is the raw sensor response data, and i provides information on the interferent factors that may help correct for both non linearities and environmental/confounding influences. f itself may be based on a physically rooted model or, more commonly, on empirical evaluations (e.g., linear models) or even black-box models shaped by sample-based knowledge (e.g., machine learning models). The latter knowledge can be gained in a laboratory-based, controlled setup or via field recordings. Aiming to characterize and calibrate the Shinyei PPD42NS particle counter in a controlled laboratory setting, Austin et al. [64] showed how the counter’s response is affected by the particle size composition, further indicating a nonlinear response curve. The authors concluded that the sensitivity of this sensor is appropriate for most outdoor locations but is not adequate for indoor scenarios where extremely high concentrations are expected, such as in smoker’s homes or in places where biomass is a source of energy for heat or cooking. It is important to remember that this sensor is a particle counter, and in cases of exposure to polydispersed aerosols of unknown composition, conversion to mass may be difficult without the availability of a concurrent gravimetric method.

Environmental interference, a very well-known issue for low cost chemical sensing, was reported by several authors. Wang et al. studied and lab-calibrated a set of LCPMS, including Shinyei PPD42NS, Samyoung DSM501A, and Sharp GP2Y1010AU0F [63]. Their environmental control setup was able to stimulate responses induced by PM and nontarget interferents. The authors found that the responses were affected by particle size distribution and composition and, to a more limited extent, temperature. Relative humidity was also found to affect the sensor responses for concentrations ranging from 0 to 1000 $\mu g/m^{3}$. The authors also concluded that all sensors showed saturation effects at 4 $\mathrm{mg}/m^{3}$ and that the noise of such sensors at low concentrations may hamper their ability to work in clean environments. Algorithm improvements, including averaging, may ameliorate performance in these scenarios.

During a very short-term deployment (8 days, 16–23 April 2013, Oakland, CA, US), Holstius et al. [90] tested several LCPMS in outdoor conditions for the first time, collocated with regulatory grade analyzers. This study included a custom Shinyei PPD42NS-based monitor and a Dylos 1700 monitor, among others. The authors proposed a field calibration strategy at two different averaging periods (1 and 24 h) for PM2.5 concentration estimation. Several Shinyei-based LCPMS were tested, showing a highly correlated response among them, with the Pearson correlation factor approaching 0.95 with the Dylos device (0.93 < r < 0.95). The correlation factor with regulatory grade data was found to be in excess of 0.74 but never reached 0.8 for the Shinyei-based devices, with results that were comparable to those of the Dylos device. Once calibrated using regulatory grade reference data, the RMSE was found to be 3.5 $\mu g/m^{3}$ for each of the tested devices, showing impressively uniform capabilities. The authors reported only a slight capability of humidity to predict the LCPMS signal, suggesting a limited influence of humidity and no influence of temperature. However, these results might be due to the limited deployment time and subsequent scarcity of high humidity events. The authors concluded that device- and site-specific calibrations could help low-cost sensors yield data of comparable quality to more costly portable nephelometers. 

Indeed, according to Reece et al. [91], high humidity events may cause catastrophic disruption to sensors’ capabilities when LCPMS are operated outdoors for long periods. Aimed at demonstrating the high spatial variance of air pollution levels, this study’s distributed deployment lasted for 5 months in Puerto Rico (Tallaboa-Encarnacion, Penuelas). The authors reported the complete or extreme loss of signals for a set of sensors (Aplhasense OPC-N2) exposed to repeated high relative humidity events. Some of these sensors never recovered until an operator power-cycled them. The authors highlighted how understanding sensor limitations is relevant for low-cost sensor deployment to advance a community’s understanding of pollutant dynamics. Shinyei PPD42NS was at the core of the study conducted by Gao et al. [92] in Xi’an, the capital of Shaanxi province in China. A network of these LCPMS-based sensor systems was deployed and colocated along with several instruments, including a TSI DustTrak II, an Airmetrics MiniVol Tactical Air Sampler, and an E-BAM instrument. The main aim of this study was to capture the expected high space–time variability across the monitored areas with different uses. The authors first calibrated the TSI DustTrack II with miniVol data and used the calibrated device as a reference for the Shinyei-based device. Based on the evidence of a nonlinear sensor response at middle to high concentrations, the authors chose a polynomial multivariate regressor (>4th grade, including temperature and humidity as predictors) for a calibrated estimation of mass concentration. The model was tuned using 4 days of TSI DustTrack II colocation data (16–20 December 2013) sampled at a 1 h maximum rate. After the calibration process, the system was able to provide performance comparable to that obtained by Holstius et al. [90]. Practically, the 24 h averaged data from the calibrated LCPMS monitors were compared to the Airmetrics MiniVol TAS data when deployed in the field at several locations (9–16 December 2013). The $R^{2}$ was found to be about 0.53 for all the LCPMS, with standard regression errors ranging from 16 to 39 $\mu g/m^{3}$. The authors noted that the devices dealt with substantially higher concentrations that regularly exceeded 300 $\mu g/m^{3}$, while the temperature and relative humidity were substantially different from those encountered during the calibration phase. The authors ultimately advocated for additional calibration studies under varying meteorological conditions in different regions but highlighted the potential usefulness of LCPMS to increase the space–time measurement resolution in health-related studies.

In 2015, Crielly et al. [93] field-evaluated the OPC-N2 sensor system. In this mid-term colocation experiment (5 months total), the performance was reported to be strongly nonlinearly affected by relative humidity events. These events triggered high water content in the sampled particles, which is expected to negatively affect OPCs. The authors applied k-Kholer theory to correct for humidity’s effects by field-calibrating the relevant parameters using field-recorded humidograms. The suggested approach indicated a significant performance improvement. This approach, however, which involves applying a correction factor to the derived PM values, is equivalent to a uniform reduction in particle number concentration over the entire particle size spectrum. In [94], instead, the authors proposed an algorithm to correct for the changes in individual particle sizes due to water uptake under high RH conditions that reflect the hygroscopic properties of real-world particles. This algorithm provides an adjusted particle size distribution that does not involve simple scaling and was used to correct the PM values estimated by the low cost OPC (Alphasense OPC-N2), thereby improving the correlation with a reference OPC (Palas Fidas200 S) over a six-week period (23 May 2017 to 31 May 2017 and 17 December 2017 to 16 January 2018). Nonetheless, the corrected algorithm parameter value depends on the particle chemical composition and, particularly, the different organic/inorganic composition ratios since these fractions show different hygroscopicity levels [95]. The authors ultimately warn the reader about possible changes to the parameter value due to seasonality effects and advocate for seasonal recalibrations. Zheng et al. (2018) [96] also reported a significant nonlinear influence of relative humidity (>70%) on several Plantower PMS3003 sensors deployed in India (Kanpur) and North Carolina (Dhuram) over several weeks of colocation (across the monsoon and post-monsoon season) using research and regulatory grade instruments. Whenever accurate and precise reference measurements were available, they showed the emergence of nonlinear behavior from the factory-calibrated sensors when the concentration values exceeded an empirically determined threshold (e.g., 125 $\mu g/m^{3}$ for PM2.5). This behavior was better accounted for by a quadratic function based on the initial linear univariate calibration. The best results, however, were obtained by empirical and nonlinear approaches correcting for humidity interference. In some cases, a limited influence of temperature was also recorded. In summary, the authors advocated the use of nonlinear multivariate field calibrations for correcting nonlinear target and nontarget (interferents) responses, demonstrating the ability of these field calibration procedures to express a relative error less than 10%.

As a counterexample, during the mid-term deployment (6-months) of several LCPMSs, Feinberg et al. reported that the RH was not a significant predictor for hourly concentration estimations obtained with Alphasense OPC-N2 sensor PM2.5, thus removing these estimations from their linear calibration functions [97]. Notably, the events that resulted in high concentration estimations (200 $\mu g/m^{3}$) were already removed from the relevant dataset based on previously reported associations with high RH events when the OPC-N2 was deemed unreliable [91,98]. Furthermore, only sensor pods that exhibited a coefficient of determination ($R^{2}$) greater than 0.5 during the colocation period (6 out of 17 sensor pods with the coefficient of determination value $R^{2}$ ranging from 0.5 to 0.81), underwent further linear regression calibrations that permitted their use in a source apportionment geospatial analysis. The authors concluded that linear recalibration was able to remove the appreciable slope and bias differences observed in several sensors.

During a nearly one-year long colocation deployment, Bulot et al. [81] reported that their LCPMS (Plantower PMS5003, Plantower PMS7003, Honeywell HPMA115S040, and Alphasense OPC-N2) performance varied with different observed PM sources and background concentrations. The deployed low-cost sensors showed general agreement with the variability measured at low PM concentrations. The authors remarked that the LCPMS might be affected differently by varying temperature and humidity and highlighted the potential need for different correction methods. The authors eventually advised against individual deployment, instead favoring redundant deployment, which may help reduce outliers and variance by averaging. In [55], the feasibility of a low-cost PM monitor based on Plantower PMS 7003 was evaluated in indoor and roadside outdoor microenvironments in colocation with TEOM (outdoor) and Grimm (indoor) reference instruments during a short-time period (2–4 days). This study focused on the linearity of the response, the precision of the measurements, and the influence of humidity using two new performance parameters. Their results show that the Plantower-based monitors work better in indoors than outdoors environment, achieving an $R^{2}=0.72-0.78$ in outdoor tests and $R^{2}=0.95-0.96$ in indoor tests for PM2.5. 

M. Jovašević-Stojanović et al. [72] calibrated a DYLOS 1700 monitor for PM10 and PM2.5 against a reference instrument (GRIMM Model 1.108 monitor) operated by the Serbian Environmental Protection Agency (SEPA) as part of the national AQ monitoring network. The authors first processed the Dylos 1700 response via smoothing algorithms and a count to mass conversion algorithm based on the literature data [99]. The authors reported an improved correlation coefficient among the reference and LCPMS sampled data due to the smoothing process. Finally, the authors used a data-driven tuning process for a first-degree polynomial calibration function, obtaining very low error rates with 1 h averaged data recordings that were usually comparable to the reference data. Based on their results, the authors concluded that appropriate data collection and processing infrastructure and quality assurance and control procedures are paramount to facilitate the wider use of such devices.

A study conducted by Hojaiji et al. analyzed and compared the performance of a Dylos DCS1100 monitor with OPC-N2 as the reference data provider for an LCPMS system assembled around a Sharp p2y1010au optical particle counter [100]. The experiments were conducted in different indoor and outdoor setups. Although short-term, the campaign offered useful insights into the influence of temperature and humidity on sensor responses by using a linear multivariate calibration (temperature and humidity correction included) procedure. Cavaliere et al. calibrated and evaluated a low-cost AQ station equipped with several gas sensors and PM sensors based on a Novasense SDS011 PM detector [57]. The calibration was conducted in a laboratory using the DustTrak DRX model 8533 as a reference instrument for PM2.5 and PM10. Then, the AQ station was placed outside the institute in a dedicated space while the same sampled air was simultaneously injected into the reference instruments through Teflon tubes. Several linear and nonlinear models were applied to the calibration procedure, and the results showed that for both sensors (PM2.5 and PM10), robust linear regression using the Talwar M-estimator was the best model. After the calibration process, the AQ system was validated on-site against a fixed AQ station ARPAT from 1 November 2016 to 15 April 2017. This validation was conducted by comparing the lab calibration with other calibration techniques. The obtained results show that the best performance for the adopted coarse 24 h resolution was obtained after a laboratory calibration of each sensor against a light-scattering meter.

Borrego et al. [101] compared several commercial and research-designed AQ multisensor devices and used machine learning approaches to calibrate the environmental gas, PM, and weather microsensors against standard reference methods through an experimental urban air quality monitoring campaign. Several calibration techniques were compared, including the linear calibration, feed forward neural network (FFNN), and random forest (RF) algorithms. For PM10, the results indicate a good overall correlation between the reference and the available measurements. A correlation coefficient of R=0.91 was the maximum value achieved with the use of RF, while a coefficient of R=0.88 was the minimum value. On the other hand, a correlation coefficient of R=0.84 was the maximum value and R=0.7 was the minimum value achieved with FFNN. For PM2.5, the results indicate that both RF and FFNN improved the correlation coefficient from R=0.53 to R=0.63 compared to the best coefficient achieved with basic calibration (ranging from R=0.36 to R=0.53). In [102], an empirically designed two-layer FFNN model was used to calibrate the low-cost PM sensors, compensating for the effect of environmental factors. The field experiment collected experimental data from 20 March to 6 May 2018 in central Taiwan. The results showed that the machine-learning method based on nonlinear multivariate regression improved the accuracy of the PM sensors from $R^{2}=0.618$ to $R^{2}=0.905$.

Short time drift effects were reported by Budde et al. when calibrating a Sharp GP2Y1010 indoors over a few days against a TSI Dust Track particle counter with a chalk dust burst generator [103]. The drift effects were then confirmed outdoors against a regulatory grade instrument. The authors proposed correcting for short term drift using repeated recalibration with higher accuracy devices for on-the-fly calibration [104,105,106]. In their experiments, the authors reported results simulating on-the-fly calibration over a limited timespan (7 days) with regulatory grade instrument data points used to correct for bias drift. Although the error results were attractive (down to 5 $\mu g/m^{3}$ for a 24 h average PM concentration estimation against regulatory data), this approach is based on very high quality calibration data that are rarely available to correct for significant short term drift. Furthermore, the timespan was too limited to draw decisive conclusions. Long term drift effects may become apparent when calibration is performed across different seasons. Zheng et al. [96] also reported an appreciable baseline drift when comparing two univariate models computed using monsoon and post-monsoon season data. However, the authors did not analyze the reasons for this effect, which may be due to a more general conceptual drift for change effects caused by the target/environment/interference (i.e., a different calibration regime), as the target concentration distribution significantly differed in the two timeframes.

In [107], the authors introduced a simultaneous Gaussian process regression (GPR) and simple linear regression pipeline to calibrate wireless LCPMS networks placed in the field on-the-fly. The network was based on Plantower PMS7003 sensors (≈USD 25; dimension: 4.8 cm × 3.7 cm × 1.2 cm) to measure PM1, PM2.5, and PM10 mass concentrations and an Adafruit DHT22 sensor to measure temperature and relative humidity. The proposed method evaluated Delhi, where 22 reference and 10 LCPMS were available from January 1, 2018 to March 31, 2018 (the global average of the 3-month mean PM2.5 among the 22 reference stations: 138 ± 31 $\mu g/m^{3}$) using leave-one-out cross validation (CV) over the 22 reference nodes. The results demonstrated that their approach can achieve excellent robustness and good accuracy, as underscored by the low variability in the GPR model 20 parameters and the model-produced calibration factors for low-cost nodes, as well as the overall 30% prediction error (equivalent to an RMSE of 33 $\mu g/m^{3}$) at a 24 h scale over the 22-fold CV. 

In [108], several LCPMS monitors based on the Shinyei PPD42NS were calibrated by colocation with a BAM-1020 particulate monitor. This setup was used for long-term measurements, which were actually started in December 2015 and ended May 2017 at Nanjing University of Information Science and Technology. Linear regression (LR), power linear regression (PLR), and artificial neural networks (ANNs) were selected as the calibration function models. The highest correlation between the calibrated low-cost sensor estimates and the BAM-1020 PM2.5 measurements (*R*^2^ = 0.84) was obtained by the ANN model, which also scored the lowest in MNB (12.66%) and MNE (29.71%). Nonetheless, unsatisfactory performance was obtained when handling low concentrations (i.e., concentrations below 35 $\mu g/m^{3}$) and very humid environments (i.e., RH > 75%) suggesting a significant influence of humidity on sensor performance. Indeed, the authors reported that the use of relative humidity and temperature as further inputs for the calibration models improved the results of the calibration methods. The authors argued that the proposed multivariate connectivity approach can be used to accurately calibrate LCPMS monitors. In the long-term, particularly starting from 18 months after the calibration, clear sensor performance degradation was observed. The authors concluded that these sensors must undergo recalibration procedures when aiming at long-term optimal performance. The authors ultimately advocated for individual calibration of the sensors. Their calibrated PM2.5 estimation models appeared to be sensor-specific, as the application of a single calibration model obtained for a specific sensor to all the other sensors yielded only moderate to poor performance.

In [109], a new combined calibration method was introduced to increase LCPMS accuracy during a field colocation experiment. Specifically, a multisensor platform was developed and colocated with the governmental BAM at the government station (Dongjak-gu, Seoul, Korea) to evaluate low-cost light scattering PM2.5 sensors (Beijing Plantower PMS7003). The data were collected for around 7.5 months (January 15, 2019 to September 4, 2019). The combined calibration method (SMART calibration) was compared to other calibration techniques, and the results showed increased correlations ($R^{2}$) between the low-cost sensors and the BAM output—specifically, 0.41 (raw signal), 0.82 (LR), 0.84 (MLR), 0.83 (MLP), and 0.89 (SMART calibration).

Most of the reviewed works relied on simple linear calibration method with mixed results that have been detailed above. Many of those, however reported nonlinear and nonselective behaviors and improved performance through the use of nonlinear or upgraded approaches like

- NNs ([101,109]);
- Random forest [101];
- Polynomial regression [72];
- Piecewise, multivariate, power, or robust linear regression ([57,96,100,108], respectively);
- GPR [107].

Similarly, nonlinear calibration models (exp, log, quad) have been found to be applied in field calibration experiments in technical reports published by AQ regulatory monitoring institutions, e.g., AQ-SPEC a lab center of South Coast AQMD in United States [66] or EPA [87]. In these reports’ focus, several complete *PM monitors* based on different LCPMS and sensor technologies are colocated in the field together with reference stations providing high valued performance assessment in real world conditions. 

Very recently, more advanced calibration models have been tested. A few months ago, Zaidan et al., have proposed and tested more modern approaches like NARX (nonlinear autoregressive with exogenous input neural networks) and even deep learning models (LSTM—Long Short Term Memory) comparing them to conventional approaches in a transfer learning setup [110]. However, they reported no performance advantage in using LSTM with respect to the NARX approach that scored the best. At the same time, Si et al. have tested the modern XGBoost approch as a data driven calibration function but again no performance advantage has been recorded in a 4 months deployment in Calgary with respect to a conventional feed forward neural network (FFNN) based calibration [111]. Finally, in September 2020, Wang et al., confirmed the validity of RF calibration as opposed to SVR and linear approaches in an attempt to correct the response of LCPMS while field deployed [112].

In summary, the available literature shows that the combined effects of interferents and hypothesized size/mass distribution dependencies make multivariate calibration a key element for the optimal operation of LCPMS. Furthermore, several authors reported nonlinear behaviors in both the target and interferent responses. The latter must be correctly accounted for using nonlinear calibration models. Multivariate nonlinear field calibrations may thus help achieve a relevant DQO by correcting ab-initio hypotheses and accounting for the influence of interferents, provided one has access to high quality data on the real concentrations and environmental conditions. However, the reviewed body of knowledge indicates that these corrections may only be valid within similar particle mass/size distribution and chemical composition conditions. The resulting sensitivity of the obtained calibration function to local effects means that the user should be careful in choosing calibration conditions that reflect those expected in the final operative scenario [106]. Despite ensuring the target and interference concentration distributions, degradation or changes in the residual sensor properties (sensor drift effects) may still negatively affect long-term performance. For this problem to be fully rectified, a network using on-the-fly continuous calibration should be applied. However, longer-term deployment experiments are still needed to ensure the performance of such devices. A final note on performance evaluation should be made. Many reviewed papers relied on *R*^2^ as a sole measure of sensor accuracy. *R*^2^ has been designed as an indicator of “goodness of fit”, and, for example, has limited capability in capturing and communicating the presence of (corrected) biases. In order to improve the community evaluation capability, a more complete set of absolute and scaled indicators should be always reported including one or more among MSE (mean squared error), RMSE (root mean squared error), CRMSE (centered root mean squared error), MAPE (mean absolute percentage error), MAE (mean absolute error).

## 5. LCPMS Basic Device Characteristics and Electronic Interfaces

In this section, we list technical and commercial information for LCPMS devices selected from among market products that have already been used and investigated by some research groups, as indicated in Table 4 above. In the Supporting Information, we include a table containing the same information but for all the sensor devices listed in Table 4, even those not yet investigated in a scientific peer-reviewed paper. 

This classification is intended to serve as an instrument to help designers find aggregated information to choose particulate sensors for both mobile and fixed installation development. At the same time, a number of LCPMS operating parameters, often referred to in commercial datasheets, are explained under possible application scenarios. 

LCPMS devices are classified by their metrological and technical characteristics obtained from the official manufacturers’ websites and freely available technical sheets. The operating parameters and technical characteristics are reported as stated in the official documents or extracted from use cases.

The metrological characteristics mainly refer to elements of LCPMS operating performance, such as the measurement range, indication errors, minimum and/or maximum particle diameter, concentration resolution, etc. Technical characteristics mainly include electric and electronic device properties but also some other device characteristics, such as the designs, weights, and dimensions than can affect specific application developments.

Notably, most of the LCPMS manufacturers below are located in Asia: three are in China (Inovafitness, Plantower, and Winsen), two are in Japan (Sharp, Shinyei), one is in the US (Honeywell), and two are in Europe (Sensirion and Alphasense).

### 5.1. Metrological Characteristics 

In Table 5, the metrological characteristics are reported. Together with the manufacturer’s name, the first column reports the device model and the reference numbers to the papers that discuss applications based on that particular sensor model. For instance, INOVAFITNESS/SDS011/ [57,67,82,113] in the first line, refers to LCPMS device model SDS011, manufactured by INOVAFITNESS (CHN), whose performance was investigated in [57,67,82].

Column two reports the device’s basic operating mechanism (which is generally related to laser-based particle scattering) along with the main application scenario, as indicated by the manufacturer or discussed in the related papers. The sensor application is, in general, related to the specific device calibration, as indicated in the “Manufacturer Calibration (or other Laboratory tests)”, where information on the calibration method is provided (reference sensors, composition of particulates, etc.), as specified in the relevant datasheets. In column 10, a summary of this information is reported. 

Column three reports the device output. Although the usual output is PM concentration for most of the devices manufactured, either in $\mu g/m^{3}$ or in pcs/L, devices developed for specific applications, such as clean room monitoring or cigarette smoking detection, can return the smoke concentration or class identification. This means that the device can provide the concentration output of small particles in the cigarette smoke separated from larger house dust via the pulse pattern of the signal output. 

Columns four, five, and six report, respectively, the measurement range and its resolution in units corresponding to the device output and the indication error, which is the relative error (%) and/or absolute error related to the device output. Notably, some devices are characterized by the possibility to further divide the measurement range into subranges for more resolved measurements.

Columns seven and eight, respectively, provide the sensor’s operating temperature range and relative humidity operating range. As also mentioned in previous sections, very low temperatures and/or high humidity levels can affect sensor performance because of possible coalescing phenomena, resulting in anomalous increases in particle size. Notably, for other gaseous interferents whose effects are known, the specific model datasheets generally provide the necessary measures to account for their influence (e.g., Sharp DN7C3CA007 [107]). Some more sophisticated sensors mount additional sensors or use more accurate internal controls (e.g., temperature, humidity, fan tachymeters, and laser power feedback) to handle data validation/correction information and sensor anomaly conditions (e.g., Alphasense N2/R1/N3 [114,115,116], EcologicSense NextPm [117], Sensirion SPS30 [118], Cubic PM2008 [119] /PM2009 [120], etc.). Since particle size is mainly determined by analyzing the amplitude (referred to as the thresholds) of an optical signal, these features can be affected by poor values of the signal-to-noise ratio as a result of limited source intensity or poor receiver sensitivity; they can also be affected by noise related to the acquisition electronic chain. The “concentration resolution" and "indication error" concentrations in the various dimensional bins of the output signal (PM1, PM2.5, and PM10 concentrations) are partly due to this effect. 

Column nine reports the minimum detectable value of the particle size for each of the sensors discussed. This value is usually determined by the wavelength of an optical signal source. Dueto the signal weaknesses near this minimum, poorer performance in particle detection can be observed, which is often described as a “counter efficiency effect” in the datasheets. 

Other effects that can result in errors in the estimation of concentrations are due to incorrect assumptions of the density of the particles related to the calibrations performed under different conditions than the actual operating ones (different types and compositions of the particulates compared to the calibration ones). Some manufacturers use various strategies to overcome this effect, such as using special algorithms to better estimate the concentration (e.g., Cubic PM3006T [121]), providing a correction parameter that is editable by the user so that ad-hoc calibrations can be implemented (e.g., Alphasense N2/R1/N3 [114,115,116], Honeywell HPMA115 series [122], Cubic PM3006T [123] /PM2009 [120], etc.), or performing multiple types of calibrations with different operating conditions and particulates, leaving the user to choose the best compromise (e.g., Plantower PMS7003 [124] /PMS1003/PMSA003 [125], Grove HM3301, Cubic PM2008 [119] /PM2009 [120], etc.). Near the upper bound of the concentration range, a typical error source is given by the proximity/shadowing phenomena in the sample volume being tested. For high values of PM concentration, the probability increases that two very close small particles will give a signal similar to a larger particle or that, vice versa, a large particle will somehow influence the pattern of a smaller one. For all sensors that automatically use separate bins for different concentrations, this effect is limited by the use of more accurate waveform processing with ad-hoc algorithms (e.g., Amphenol SM-PWM-01C, Plantower PMS7003 [124], etc.).

As discussed above, factory calibration operates directly on the raw sensor response, generally obtaining a calibration curve for a given specific application. The possibility to access raw data is, therefore, of great relevance for investigating sensor performance in applications different from those indicated by the manufacturer or when different calibrations are applied. For each model, column 11 reports the possibility to access the raw data.

**Table 5:** Metrological characteristics, obtained from the official manufacturers’ websites and freely available technical sheets. The first column refers to the manufacturer’s name, the device model, and the papers and refers to applications based on that sensor model. Column two reports the device’s basic operating mechanism and its main application scenario. Column three reports the device output in μg/m^3^ or pcs/L. Columns four, five, and six report, respectively, the measurement range and its resolution in units corresponding to the device output, as well as the indication error related to the device output. Columns seven and eight are, respectively, the sensor operating temperature range and the RH operating range, respectively. Column nine reports the minimum detectable value of the particle size for each of the sensors discussed. Column 10 reports information on device calibration and the way it was performed. In column 11, the possibility to access raw data is shown.

Manufacturer/Model/Ref	Type/Main Application	Main Measured Output Data	Measurement Range (µg/m^3^)	Concentration Resolution (µg/m^3^)	Working Temperature Range (°C)	Working Humidity Range (%RH Noncondensing)	Error	Particle Diameter Resolution or Range (µm)	Manufacturer Calibration (or Other Laboratory Tests)	Raw Data Availability
Alphasense/OPC/N2/ [81,82,93,94,97,98,114,126,127,128]	Particulate monitor/outdoor	PM1, PM2.5, and PM10	10000 (particles/second)	0.01	−20 to +50	0–95%	NA	0.38–17	Method defined by European Standard EN 481/TSI3330-GRIMM1.108 comparison	16 bins/1.4 to 10 μm/modifiable particle density value
Honeywell/HPMA115S0-XXX/ [67,122,129,130]	Laser-based light scattering particle sensing/indoor-automotive	PM2.5 PM10	0–1000	1	−20 to +50	0–95%	PM2.5: 0–100 ± 15 µg/m^3^ PM2.5: 100–1000 µg/m^3^ ± 15%	NA	NA	Customer adjustment coefficient
Inovafitness/SDS011/ [57,67,82,113]	Laser based PM2.5;10 sensor/indoor	PM2.5, PM10	0–1000	0.3	−10 to +50	0–70%	Maximum between ± 15% and ± 10 μg/m^3^	0.3–10	NA	NO
Plantower/PMS 1003/ [80,131]	Laser based particle concentration sensor/indoor	PM 1, PM2.5, PM10	0–500	1	−10 to +60	0–99%	100-500 μg/m^3^ ± 10% 0–100 ± 10 μg/m^3^	0.3	Standard particles/atmospheric environment	6 bin particle number; standard particles concentration atmospheric environment concentration
Plantower/PMS 7003/ [55,81,82,124,129]	Laser based particle concentration sensor/indoor	PM 1, PM2.5, PM10	0–500	1	−10 to +60	0–99%	100–500 μg/m^3^ ± 10% 0–100 ± 10 μg/m^3^	0.3	Standard particles/atmospheric environment	6 bins particle number; standard particles conc/atmospheric environment conc
Plantower/PMS A003/ [77,125]	Laser based particle concentration sensor/indoor	PM 1, PM2.5, PM10	0–500	1	−10 to +60	0–99%	100–500 μg/m^3^ ± 10% 0–100 ± 10 μg/m^3^	0.3	Standard particles/atmospheric environment	6 bin particle number; standard particles conc/atmospheric environment conc
Sensirion/SPS30/ [118,132]	Particulate matter sensor/indoor–outdoor	PM1.0, PM2.5, PM4, PM10	0–1000	1	10 to +40	20–80%	PM1, PM2.5: 0–100 ± 10 μg/m^3^ 100–1000 μg/m^3^ ± 10% PM4, PM10: 0–100 ± 25 μg/m^3^ 100–1000 μg/m^3^ ± 25%	0.3	PM2.5 mass concentration calibrated to TSI DustTrak™ DRX 8533 Ambient Mode PM2.5 number concentration calibrated to TSI OPS 3330	5 bin particle number
Sharp/GP2Y1010AU0F/ [63,65,67,83,133,134,135,136]	Led based dust sensor/indoor	PM10	0–500	Noise lim	−10 to +65	NA	NA	NA	Cigarette smoke reference: dust monitor (P-5L2: manufactured by SHIBATA SCIENTIFIC TECHNOLOGY LTD)	NA
Shinyei/PMS1/ [98,137]	Particulate sensor/-	PM 2.5	0–200	NA	−10 to +45	20–85%	NA	0.3	NA	NA
Shinyei/PPD20V/ [138,139]	/indoor	PM10 (pcs/liter)	0–30,000 (pcs/l)	NA	0 to +40	0–95%	NA	1	Cigarette smoke, concentration reference: Rion Kc01/drop test, vibration, high temperature and humidity endurance	Yes
Shinyei/PPD42NJ/ [63,138,140]	Particle sensor unit/indoor	PM2.5, PM10	0–8000 (pcs/283 ml = 0.01cf)	NA	0 to +45	0–95%	NA	1	Cigarette smoke, weight concentration reference: sibata LD5 reference, concentration reference: Rion Kc01/drop test, vibration, high temperature and humidity endurance,	NA
Shinyei/PPD60PV-T2/ [139,141]	Particulate sensor/indoor	PM10 (pcs/l) detects air borne particles from cleanliness class 100000–1000000	0–20000 (pcs/283 mL(0.01 cf) (0.5 um range particle))	NA	0 to +45	0–95%	NA	0.5	Cigarette smoke, concentration reference: Rion Kc01/drop test, vibration, high temperature and humidity endurance	Yes
Winsen/ZH03/ZH03A/ZH03B/ [67,82,142]	Particle sensor PM2.5 dust sensor/indoor	PM1.0, PM2.5, PM10	0-1000	NA	−10 to +50	0–85%	NA	0.3	NA	NA

### 5.2. Technical Characteristics

Table 6 reports the technical characteristics of LCPMS, a group of parameters that can be used as a reference to optimize the design of the monitoring system based on the application scenario.

The first column reports, as in Table 5, the device model, the manufacturer’s name, and the numbers related to the papers that discuss applications based on that sensor model. 

The second column refers to the dimension and weight of the LCPMS. In many applications where the sensor must be compact and light (i.e., IoT portable devices), this factor is a key issue, while in other applications, such as fixed measurement stations, dimension and weight are often less relevant. For all the devices listed, the device volumes range from around 20 cm^3^ (the Plantower and Sensirion families) to about 150 cm^3^ for the Shinyei family. The weight ranges from 16 g for Sharp/GP2Y1010AU0F [136] to 130 g for Shinyei [137].

The third column is related to the device’s power supply and shows the power input required to properly supply the sensor. Depending on the application, the designer should choose a sensor while taking into consideration the other devices present on the acquisition board and all the operating voltages required, included that of the sensor.

In the fourth column, the maximum absorption of current during normal device operation is reported. This parameter is useful for two reasons: the designer can use it estimate the overall absorption of the acquisition system (especially in presence of low power constraints), and by knowing the correct operating conditions of the sensor, it is possible to properly dimension the protection devices of the electronic board and quickly detect any failures due to the sensor. 

The fifth column reports the current in mA consumed by the sensor while in sleep mode (if applicable). Typically, in low power applications, it is useful to put the sensor into a low power state when it is not in use. Some sensors also allow one to activate a low power acquisition mode; the notes in this column also report this possibility. 

The sixth column reports the possibility to modify the laser power absorption, providing a chance for the designer to tune the power consumption of the entire system. For many applications, especially those related to IoT, metering devices are powered by batteries. Thus, a reduction of energy consumption is a powerful tool to ensure a reasonable operational lifetime of a system. Of course, when laser power regulation is active, the measurement output will, in general, depend on the laser intensity.

The seventh column reports the “response/warm up time” for the various devices listed. This parameter indicates the minimum time required by the sensor to give a response after a query. Some of the sensors may introduce some latency into the response due to on-sensor elaboration, whose knowledge is required to properly select the sampling frequency. Indeed, latency can impose limits on the maximum sampling frequency that the designer can select, but in the majority of applications investigated so far, the sampling time is not this critical. The warm up time field is mainly affected by the laser startup, that is, the time required by the laser to become stable enough to properly work and the time the fan (if any) needs to reach its working speed.

The eighth column reports information related to the “Output interface (Bus type)/Level”. When in the operating mode, the information related to the number of suspended particles in the air needs to be transferred and further processed by other components of the monitoring system to obtain meaningful information. Each sensor has its own interface, either an analog output or a digital bus, which can transfer data to a microcontroller. Knowledge of this interface type is important to select an acquisition board or vice-versa. In practical applications, when the sensor outputs a digital number representative of the measurement, the sensor itself includes an embedded analog to digital converter. This type of converter is useful because it avoids the need for a further acquisition device on the control board but, on the other hand, generally suggests that the ADC resolution cannot be modified. If the device output is analog, the designer has more flexibility in choosing the resolution of the ADC and using some electronics useful to condition the analog output signal. The designer can also apply oversampling techniques to reduce the noise, improve signal resolution, and optimize the final measurement errors and resolution of the acquired signal. The “Level” section shows the voltage levels available for the physical layer of the serial interface of the sensor.

The column “Flow type/inlet-outlet position” indicates how sampled air enters into the detection chamber. The way the air is sampled affects the quality of the measurement since the particle concentration is computed while relying on the volume of the sample of air being examined. Some sensors are passive in the sense that they simply let air freely flow into the detection chamber (Cubic PM1006K [143], Sharp GP2Y1010AU0F [137]). Other sensors use heaters to induce forced air convection (Amphenol SM-PWM-01C [144] /SM-PWM-01S [145], Cubic PM1003 [146], Panasonic PM2.5 Sensor [147], SAMYOUNG S&C PMSU [148] /PMSL [148], Shinyei PPD series ([139,140,141,149]), and Winsen ZPH01 [150] /ZPH02 [142]). Still others use a fan that forces a constant air flow to enter into the detection chamber (Alphasense OPC N2/R1/N3 [114,115,116], Amphenol SM-UART-01D [151] /SM-UART-01L+ [152] /SM-UART-04L [153], Bjhike HK-A5 [154], Cubic PM2008 [119] /2008M [155] /2009 [120] /PM2107 [156] /PM2105M [157] /PM2012 [158] /PM3015 [159] /PM3006T [160] /PM5000 [161], Grove HM3301 [162], Honeywell HPM Series [122], NanoSensePM2036 [163], Inovafitness SDS011 [113] /SDS018 [164], Panasonic SN-GCJA5 [165], Plantower PMS7003 [124] /1003 [131] /A003 [125], Sensirion SPS30 [118], Sharp DN7C3CA007 [166], Shinyei PMS1 [137], TianjinFigaro-isweek TF-LP01 [167], Winsen ZH06 [168] /ZH03 [142], and YAGUCHI ELECTRIC CORP. SDS021 [169]). In the latter case, the sensor results are less affected by possible environmental effects. Furthermore, when the fan is associated with a tachymeter (or another equivalent flow control tool), the accuracy of the measurement is considerably increased.

The 10th column, “Lifetime/ageing phenomena”, reports data related to the device’s lifetime reliability to highlight information related to system maintenance.

The last column, “Approximate Cost range”, shows the price range of the devices (an approximate synthetic parameter) at the time of this review.

### 5.3. Applications

Considering the information provided by the datasheets and listed in Table 5 and Table 6 (as well as in the extended Table reported in the SI), the reviewed LCPMS models can be classified by their suitability under four general application scenarios:

1. IoT distributed applications. These applications require the use of devices that have low average consumption during the acquisition phase, can be placed into a low power state, and allow the regulation of the intensity of the laser. Depending on the application, the devices in this group can match the low power requirements if properly managed.
2. Sensor flexibility. Some applications require the device to operate under conditions that could differ from those used to calibrate the sensor. This group includes devices that are claimed to be endowed with one or more of the following characteristics: high quality factory calibration, the ability to regulate the calibration, the availability of raw data, and a number of bins for the output data.
3. On board integration complexity. This parameter is related to the complexity of the integration process of the sensor on a custom acquisition board or equipment. For example, some sensors require the output signal to be conditioned by custom hardware, while others are provided with embedded signal conditioning hardware. In the former case, the designer has more flexibility, but additional hardware is required; in the latter, less effort is required for the integration of the device on the acquisition board. It is also important to choose a proper sensor while considering the environment in which it will be deployed since some factors can negatively affect the measurements, such as droplets, fog, vibrations, wind, direct light, dew, temperature, humidity, soot, grit, air-flow rate obstruction, the mounting environment, the mounting position, and orientation. In this area, the availability of advanced sensor cases is often a key issue. In general, the main parameters required to choose a sensor in this group are the presence of a fan, the presence of signal conditioning hardware, case completeness, factory calibration, and additional onboard sensors (e.g., temperature/humidity sensors and a fan tachometer).
4. Applications that require advanced metrological properties. This group includes sensors that can provide thorough information on measurement errors, resolution, and the range of concentrations that can be detected.

In Table 7, the reviewed LCPMS are grouped according to the four application scenarios defined above for the devices listed in Table 4.

## 6. LCPMS Performance Literary Review

### 6.1. Methodology

This section reports the performance exhibited by the LCPMS that were investigated in the scientific literature (Scopus, keywords: (particulate AND matter OR PM) AND (low-cost OR personal OR pervasive OR sensor *) in “Title”). The sensors are classified by a number of characteristic parameters, mostly derived from the EPA definitions (see Section 3) and more extensively reported in the SI.

Some of the most meaningful parameters are summarized in Table 8, where the columns refer to the quality indicators which were reported by authors to evaluate the performance of their sensing apparatus in comparison with a reference instrument, which is reported in the last column.

- **Accuracy**: A measure of the overall agreement of a measurement with a known value (i.e., an accepted reference value). Along with bias, the *R*^2^ coefficient of a regression model predictions, hereby listed, is a generally accepted measure of the calibrated instrument potential accuracy. Its value may range from -∞ to 1.
- **Precision**: A measure of the agreement among repeated measurements of the same property under identical or substantially similar conditions, calculated either as the range or as the standard deviation.
- **Bias**: The systematic or persistent distortion of a measurement process that causes errors in one direction.
- **Completeness**: A measure of the amount of the valid data that needs to be obtained from a measurement system.
- **Detection limit**: The lowest analyte level that can be confidently identified.
- **Measurement range**: The minimum to maximum concentration range that the instrument is capable of measuring.

When different reference instruments are used, a direct comparison among reported performance levels requires caution. Some works compare the concentration estimations of low-cost devices with those of measurement methods that are officially recognized by government agencies, i.e., those based on different measurements principles such as FRM and FEM, as discussed in Section 2. A relevant example is found in the work of Sayahi et al. [80], who tested two LCPMS produced by Plantower with a gravimetric FRM and a TEOM (FEM) over a period of 320 days. Due to the long sampling time of FRM and FEM, the comparison was performed by considering the average of the measurement periods that became comparable to those of the reference instruments. In other investigations, LCPMS were compared to certified OPCs used as reference instruments. These comparisons were implemented mainly under laboratory conditions with controlled setups, although several papers report field deployment performance. The high temporal resolution of OPCs, which is comparable to that of LCPMS, provides more accurate information on the quality of the measurements from low-cost devices. For example, Feinberg et al. [98] compared the performance of LCPMS with a Grimm 180 EDM, and Han et al. [172] used a Grimm 11R as a certified tool for comparing devices over a duration of 12 days.

Several works rely on both types of reference tools to achieve an even broader comparison. For example, Crilley et al. [93], in addition to a TEOM (FEM) used two certified OPCs (a TSI3330 and a Grimm Pas1.108) as reference tools, and Mukherjee et al. [126] used a BAM- 1020 (FEM) and an OPC Grimm-11R.

### 6.2. Performance Review Results

Below we summarize the results for the most representative LCPMS families.

#### 6.2.1. Alphasense N2

As shown in Table 7, this is the PM sensor most commonly tested by the scientific community. Among low cost sensors, this is one of most expensive and is able to measure PM1, PM2.5, and PM10 concentrations since it can count particles with dimensions between 0.3 and 25 µm, dividing this range into 16 dimensional bins.

Crilley et al. tested this sensor through field evaluations compared against two different reference systems for a period of two weeks [93]. The two reference systems were a Grimm PAS-1.108, which is a certified optical particle counter, and a TEOM, which is an FEM recognized by the EPA. In this work, the authors discuss issues related to humidity interference, highlighting that for all the sampling points obtained over the two weeks, the *R*^2^ values reached 0.72 for both the Grimm and the TEOM when measuring PM2.5, with 0.67 for the TEOM and 0.68 for the Grimm when measuring PM10. However, these correlation coefficients increased significantly, in all cases exceeding 0.8, when only sampling points with measured relative humidity lower than 85% were taken into account. This confirms that humidity is one of the most important interferents for this kind of device.

Jhonston et al., as part of an IoT project, aimed at creating an Air Quality Monitor with the use of an LCPMS. The authors tested various sensors in the field, particularly in the surroundings of two schools, over two weeks in June 2018 [129]. The authors compared their data with data from the AURN stations, which is the official reference method used in the UK. For PM2.5 measurements, Alphasense N2 obtained the worst *R*^2^ of 0.5, compared to the values greater than 0.7 measured on other LCPMS, such as Plantower PMS 5003, Plantower PMS 7003, and Honeywell HPMA115S0.

Additionally, Badura et al. [82] tested this LCPMS in the field together with three other devices, thereby comparing them with a TEOM over a 6 month measurement campaign. The novelty of this work is its comparison between the LCPMS and the reference instrument using different averaging times (1 min, 15 min, 1 h, and 24 h). As expected, better results were obtained for all devices when increasing the sampling time. As in [98], the N2 provided the worst results, reaching a maximum $R^{2}$ value of 0.69.

In [98], Feinberg et al. tested several environmental sensors in the field, not only for PM but also for gases, over a period of 6 months between the end of 2017 and the beginning of 2018. The authors used a Grimm EDM180 as a reference for PM, with a sampling time of 1 min. In this work, the authors obtained the worst overall result for the Alphasense N2, reaching only 0.2 for the correlation index $R^{2}$ for PM2.5and 0.46 for PM10.

Mukherjee et al., in a work describing a 3-month campaign in the field, estimated the effects of wind on the measurement of particulate matter using these devices [126]. The authors used two different reference devices, one with the same physical principles as the LCPMS, the Grimm-11R, and the other with a different physical principle, the BAM-1020 (FEM). As result, they obtained an *R*^2^ equal to 0.84 for the PM10, indicating good performance, but the *R*^2^ dropped dramatically to 0.43 in the best case, compared to the Grimm, for PM2.5.

Bulot et al. tested four LCPMS sensors, including an Alphasense N2, over two months in two schools in the UK, using the official government system as a reference (AURN) [81]. Among the sensors tested, N2 again offered the worst performance for PM2.5 with a maximum correlation coefficient of 0.45. This value is very low compared to that obtained using Plantower, which exceeded 0.7 for *R*^2^.

In [97], Feinberg et al. compared 20 different units of Alphasense OPC-N2 with a TEOM for about 6 months. In this work, the *R*^2^ values of the various units were very scattered, reaching a maximum of 0.81 but below 0.5 for most units.

Di Antonio et al. calibrated the N2 in the field using as a Palas Fidas 200 as a reference. This reference device is a certified instrument based on optical light scattering [94]. The authors showed that, after the calibration, the *R*^2^ increased from a value of 0.34 to a much more acceptable value of 0.78 by correcting the data based on the relative humidity recorded. This once again confirmed that this device suffers under particularly high humidity levels.

In [127], Sousan et al. calibrated their sensor in a laboratory and not in the field (i.e., they used a controlled environment with a particulate concentration up to 10,000 $\mu g/m^{3}$). This value is much larger than the dust regimes found in the field, which barely exceed 100 $\mu g/m^{3}$. In this case, the *R*^2^ reached 0.99 for both PM10 and PM2.5. The authors concluded that at high concentrations and under controlled conditions, the performance of this device is excellent.

Finally, despite its price (which is the highest among the LCPMS), N2 provides the worst performance in the field, as indicated in the literature. N2 is one of the first LCPM sensors that appeared on the market and was provided with a detailed datasheet reporting relevant laboratory tests. Its strong points include its hardware and firmware, which allow one to gather detailed information not only on PM measurements but also on the particle bin counts and their specificity for PM2.5. However, N2 underperforms in low concentration ranges and is one of the most bulky devices.

#### 6.2.2. Plantower Family

Plantower (models PMS1003, PMS3003, PMS5003, PMS7003, and PMS-A003) offers a large variety of sensors with a lower price than other manufacturers.

Zamora et al. tested the PMS-A003 both in the laboratory under an internal environment and in the field, comparing the data with a gravimetric reference instrument, the pDR-1200, for the evaluation of PM2.5 [76]. In the laboratory, the correlation coefficient was 0.97 when using incense fumes as a source of particulate matter. For the indoor environment, the *R*^2^ was 0.92 when using kitchen smoke, while for the outdoor evaluation, the *R*^2^ was 0.91 and reached 0.93 when the sensor was calibrated. Moreover, the influences of temperature and humidity were corrected.

In [80], Sayahi et al. tested both PMS1003 and PMS5003 in the field with a 320-day campaign in which a gravimetric FRM with a 24-h sampling period and a TEOM (FEM) with a 1-h sampling period were used as reference systems. The authors sorted the results by different seasons and showed that the results were generally much worse in the spring than in the other seasons. The authors achieved the best results in winter, obtaining an *R*^2^ of 0.97 for PM2.5 with both devices. During the winter, for PM10, the authors also found a correlation coefficient of 0.91 for the PMS1003, and 0.7 for the 5003. When comparing 1 h sampling data (i.e., the TEOM results with LCPMS results for PM2.5), the Plantower devices showed comparable performance, reaching 0.88 for 1003 and 0.89 for 5003.

Wang et al. [55] tested the recently produced 7003 sensor by Plantower in the field. The authors evaluated the performance for both PM10 and PM2.5 using two different reference devices, a TEOM and an OPC (the GRIMM 1.108). For TEOM, the correlation coefficient for PM2.5 was 0.78, while that for PM10 was 0.73. These values grew significantly, both exceeding 0.96, compared to the OPC, which is based on the same physical principles of the sensor under evaluation.

Compared with the AURN control units, in [129], the correlation coefficients for two sensors of this family (PMS5003 and PMS7003) for PM2.5 were 0.76 and 0.73, respectively.

Along with the Alphasense N2, Badura et al. [82] field-tested the PMS7003 and evaluated the responses at different sampling times. The correlation results for the PM2.5 measurements yielded an *R*^2^ of 0.69 for the Alphasense N2 and 0.93 for the PMS7003, calculated with respect to a 1400a TEOM.

In [81], two devices of the Plantower family, the PMS5003 and the PMS7003, were tested in the field using an AURN station as a reference. From the comparison of PM2.5 the authors obtained correlation coefficients of 0.7 and 0.77.

To summarize, this review focused on Plantower sensors. Despite their cheaper price, these sensors provide good performance and good quality fabrication.

#### 6.2.3. Novasense SDS011

Another notable competitor is Novasense SDS011, a meter for PM2.5 and PM10 with an affordable cost of around USD 20.

In [57], Cavaliere et al., after calibrating their device in the laboratory (there device was inserted into an IoT infrastructure together with other gas sensors for complete monitoring of environmental pollutants), compared their results with an ARPAT control unit (the official measurement method in Italy) over a campaign that lasted about 6 months. This device exhibited excellent performance, providing *R*^2^ values of 0.96 and 0.91 for PM2.5 and PM10, respectively.

Badura et al. (who tested the Plantower PMS7003 and the ALphasense OPC-N2), also tested a Novasense device [82]. This device provided performance comparable with the Plantower, reaching a correlation coefficient of 0.90 for PM2.5.

Hapidin et al. tested their sensor in a laboratory in a special chamber for the evaluation of low cost devices to measure particulates [67]. In these conditions, the Novasense device provided an *R*^2^ of 0.994 for PM2.5.

Despite the small number of scientific works examining the Novasense SDS011, the consistency of the results among the various tests seems to indicate that this device is one of the best performing options in the laboratory and in field tests.

#### 6.2.4. Sharp GPD2y1010AU0F

In [133], Li et al. compared the Sharp GPD2y1010AU0F with a TSI SidePark in a carpentry shop to evaluate its performance in the workplace by inserting the sensor itself into an IoT infrastructure with the support of an Arduino and a RaspberryPi. In an indoor carpentry shop where the concentration of dust was high, the sensor obtained a correlation of 0.99 using the reference instrument.

Marinov et al. tested a battery of these sensors in the laboratory using the Alphasense OPC-N2 as a reference device [134]. High concentrations reached 1000 $\mu g/m^{3}$; for the best performance, the correlation index was 0.99.

Wang et al. also tested this device in the laboratory [55]. Referring to an OPC certified by a TSI SidePark, this sensor reached a correlation coefficient very close to 1 for PM2.5.

In [65], Sousan et al. performed a laboratory test for this sensor, finding results in agreement with the previous ones and a correlation coefficient of 0.95.

Olivares et al. tested this sensor together with a Sidepark in a house to measure PM10 [135]. The authors’ aim was to evaluate whether these sensors are sensitive to movementi.e., the movement of dust caused by human movement. Even when keeping the concentrations very low (around 3 $\mu g/m^{3}$), the correlation coefficient for a sampling time of 90 min was 0.99.

Alvarado et al. installed the sensors for environmental monitoring (including the Sharp) on a drone [83]. Before completing the infrastructure, the authors tested the sensors in the laboratory and compared them to a TSI Dusttrak 8520, obtaining 0.98 and 0.91 as correlation coefficients for PM2.5 and PM10 respectively.

#### 6.2.5. Shinyei Family

Another family of low-cost particulate sensors is produced by Shinyei: the PMS-SYS-1, PPD42NS, PPD20V, and PPD60PV models. Feinberg et al. compared the first sensor in this list to a Grimm EDM 180 in the field [98]. In this case, the sensor provided an *R*^2^ of 0.52.

The other three sensors were tested together by Jhonson et al. [138]. The test was performed in laboratory conditions using a DustTrak TSI as a reference instrument and keeping the concentration below 500 $\mu g/m^{3}$. The authors obtained correlation coefficients of 0.8, 0.98, and 0.97 for PPD42NS, PPD20V, and PPD60PV respectively.

#### 6.2.6. Other Sensors

In addition to the sensors previously discussed, there are many others that have attracted much less interest by the scientific community. The Honeywell HPMA115S0, for example, was tested only for PM2.5 [67,129,130]. In [67], laboratory tests showed that Honeywell could reach correlation coefficients as high as 0.99. In [129], field tests provided an *R*^2^ of 0.77, which was higher than that provided by an Alphasense N2 and only slightly lower than the correlation performance of two sensors from the Plantower family. In [130], a field-test was performed using a Grimm EDM 180, achieving an *R*^2^ of 0.58 over a 13-day campaign.

Another sensor that fits into this class of devices is the Winsen ZH03A, which was tested in the field by Badura [82]. The comparison in the field provided and *R*^2^ value of 0.81 but a value close to 1 in the laboratory, which is agrees with the results for all the other devices.

The Sensirion SPS30 is a relatively new sensor, with its production having started in 2018. Its peculiarities include an ultra slim package and a HEPA filter to avoid performance degradation due to powder build-up. The only scientific work that investigated its performance showed that this sensor is not suitable for PM10 measurements [132].

The last sensor included in this list is the Samyoung DSM501A, which is evaluated under laboratory conditions in [63]. The results reported showed a very high correlation coefficient of 0.98.

From all these articles, it is possible to outline some useful rules that can be adopted for the usage of these low cost instruments:

- Relative humidity is a crucial environmental parameter, and keeping the humidity lower than 85% is important to avoid a rapid degradation in accuracy
- Using high sampling times and averaging the data increase the accuracy of PM measurements, especially at low PM concentrations ( 30 μg for PM2.5), where LCPMS suffers from the worst accuracy.
- All LCPMS sensors showed the best performance with PM 2.5.
- The default calibration for an LCPMS is only a recommendation and provides good accuracy only under restricted conditions.
- Within the same brand and model of LCPMS, the quality parameters can vary. Therefore, a laboratory test is mandatory to verify the quality parameters for each sensor.
- Specific seasonal calibrations in the field are necessary to achieve the best performance, despite changes in PM typology and humidity interference.

## 7. Discussion and Conclusions

As stated in Section 1, the interest in LCPMS devices is increasing and strongly supported by the demand for the more direct role of citizens in monitoring their lived environments. Most recently, the research on the health effects of particulate matter has focused on lower particle size fractions (i.e., <1 µm), which has boosted research on innovative detection devices. The current reference methods, despite providing the most complete and reliable assessments of PM pollution, are not able to respond to the growing demand for real-time and diffuse information. Even the reference methods that allow for hourly measurements (such as TEOM, BAM, etc.) require equipment to be integrated in bulky, fixed monitoring stations, which necessarily cover a limited spatial region. Moreover, this reference equipment does not allow the monitoring of indoor environments, which are often polluted by ultrafine particles to a much greater extent than outdoor environments. This is why an ever-increasing number of LCPMS models are being commercialized (totally almost 50 types, as reviewed in this paper), mostly by companies located in Asia.

In Section 3, we briefly reported on the Mie theory underlying LCPMS’s principles of operation. This theoretical treatment helps, in practice, to direct the choice of light sources in the visible and near infrared spectra for the detection of particles in the range of 0.1–100 µm to guarantee the validity of direct proportionality between the value of scattered light intensity and the particle diameters. The particle size distribution is determined by comparing the light intensities with a standard curve, calibrated using a set of uniform particles with known diameters; therefore, assuming a spherical geometry and a mass density of 1 g/cm^3^, the diameters of the particles can be converted into mass measurements.

In Table 5 and Table 6, we summarized the metrological and technical characteristics of most LCPMS devices, selected from among the market products that have been already investigated by various research groups. These tables are mainly intended as an instrument for designers to find aggregated information that can facilitate the choice of particulate sensors for both mobile and fixed installation development. The Supporting Information section completes this review by also considering sensor devices not yet investigated in scientific peer reviewed papers. All the above information was then reclassified by considering application scenarios characterized by four specific fingerprints: output flexibility, integration complexity, IoT, and advanced metrological properties. This information is generally the most useful but is also generally missing, as stressed in a recent review [172]. Section 6, on the other hand, was fully devoted to reviewing the current scientific literature discussing the listed LCPMS devices. In general, as summarized in Table 8, these kinds of devices follow the trends of PM changes in the environment and also exhibit good performance with accuracy that, under some conditions, can reach *R*^2^ values as high as 0.99. However, such results strongly depend on whether or not the device is calibrated using some FEM or FRM methods in the operative environment. If not, *R*^2^ values lower than 0.5 are commonly observed.

Field calibration is a very critical factor for this class of devices. The vast majority of field calibration literature aiming to improve accuracy in medium- to long-term field deployments reported interference (mostly from high humidity levels) issues and highlighted several nonlinearities, as reviewed in Section 4. Almost all authors agreed with the need for multivariate regression approaches to calibrate LCPMS devices, thereby solving these issues and correcting their impacts. Some authors call for the use of nonlinear regression to cope with the observed nonlinear behaviors. Field calibration may indeed provide a solution to match composition in calibration and operational regimes. However, the same dependence of the LCPMS’s sensitivity to particulate composition and weather conditions warns against generalizing the results obtained by specific field-data-driven calibration, especially when deployments in different locations are considered. To rule out possible sensor aging or fouling, as well as composition or weather mismatches, ongoing recalibrations using high quality model data or communication with a high accuracy station is suggested.

Related to the field calibration issue is the assessment of laboratory characterization procedures. In the lab, LCPMS performance is usually evaluated inside an environmental chamber where physical parameters such as temperature and relative humidity are controlled. In some cases, other gases are also considered. Different labs may use different aerosol sources, such as cigarette smoke, incense smoke, or even welding fumes. Alternatively, to create a uniform aerosol in terms of concentration and composition, some authors use completely automated aerosol generators. In general, sensor outputs are compared to various reference instruments, and important parameters—such as the linear correlation coefficient, the detection limit, temperature, and RH effects—are recorded. Nevertheless, the PM sensor data can result from PM concentration decay over time or the introduction of a forced air flow to quench the process. The PM aerosol may also be maintained in the flow at wind speed. Linearity, calibration curve, and precision are the basic characteristics used to evaluate the reliability of PM sensors in the laboratory. The limits imposed on the chamber size by the chemical–physical variability of particulate composition due to the effect of interferents and weather conditions clearly indicate that lab characterization, although necessary, cannot replace PM sensor calibration in the field.

## Figures and Tables

**Table 1:** Low-cost PM sensor node applications, both indoor and outdoor, extracted from the scientific literature investigations and web searches.

Indoor	Outdoor
Concentration range up to thousands of μg/m^3^	Concentration range of 500 μg/m^3^
➢ Air purifiers ➢ Air cleaners ➢ Air quality monitors ➢ Air conditioners ➢ Ventilation systems ➢ Smoke fire alarms ➢ Consumer electronic products ➢ Environmental monitors ➢ Household air purifiers ➢ Automobile air purifiers ➢ Residential and commercial air conditioning ➢ HVAC systems ➢ IoT hardware intelligence ➢ Automotive applications ➢ All kinds of IAQ monitors ➢ Checking pollution “hotspots” ➢ Personal exposure monitoring	➢ Outdoor air quality monitoring ➢ Emissions monitoring ➢ Construction site monitoring ➢ Mining site monitoring ➢ Port and bulk handling terminals ➢ Fence line monitoring ➢ Brownfield developments ➢ IoT hardware intelligence ➢ Checking pollution “hotspots” ➢ Personal exposure monitoring

**Table 2:** Current exposure standards to PM10 and PM 2.5 in Western and Eastern countries.

	Averaging Time	EU ^a^	U.S. ^b^	China ^c^	Hong Kong ^d^	Japan ^e^	Taiwan ^f^	Australia ^g^	WHO Guideline Values ^h^
PM10 μg/m^3^	24 h	50	150	150	100	100	125	50	50
	Annual	40	-	70	50	-	65	25	20
PM2.5 μg/m^3^	24 h	-	35	75	75	35	35	25	25
	Annual	25	12	35	35	15	15	8	10

^a^ 2008/50/EC Directive on Ambient Air Quality and Cleaner Air for Europe. ^b^ https://www.epa.gov/pm-pollution/table-historical-particulate-matter-pm-national-ambient-air-quality-standards-naaqs, retrieved 10 October 2019. ^c^ http://kjs.mep.gov.cn/hjbhbz/bzwb/dqhjbh/dqhjzlbz/201203/W020120410330232398521.pdf. ^d^ “Air Quality Objectives”. Environmental Protection Department, Hong Kong. 19 December 2012. Retrieved 27 July 2013. Retrieved 10 October 2019. ^e^ https://www.env.go.jp/en/air/aq/aq.html, retrieved 10 October 2019. ^f^ https://taqm.epa.gov.tw/taqm/en/b0206.aspx, retrieved 10 October 2019. ^g^ Department of the Environment (25 February 2016). “National Environment Protection (Ambient Air Quality) Measure”. Federal Register of Legislation. Retrieved 10 October 2019. ^h^ https://www.who.int/news-room/fact-sheets/detail/ambient-(outdoor)-air-quality-and-health, retrieved 10 October 2019.

**Table 3:** The most common sources of PM with the types of related PM emitted. Data are adapted from [47].

	Source		PM Size	
	Beech burning	PM10	TSP	
	Hard wood burning	PM10	TSP	
	Larch burning	PM10	TSP	
	Leaves burning	PM10	TSP	
Biomass burning	Oak burning	PM10		
	Olive oil burning	PM10		PM2.5
	Pellet burning	PM10	TSP	
	Natural gas burning	PM10	TSP	PM2.5
	Wood burning	PM10	TSP	PM2.5
	Coal burning	PM10	TSP	PM2.5
Fossil fuels	Coke burning	PM10	TSP	PM2.5
	Boiler	PM10	TSP	PM2.5
	Refineries		PM10	
	Ammonium nitrate		PM10	PM2.5
	Ammonium sulfate		PM10	PM2.5
	Iron and steel prod.	PM10	TSP	PM2.5
Industrial	Metal smelting		PM10	PM2.5
	Fertilizer prod.		PM10	PM2.5
	Cement	PM10	TSP	PM2.5
	Ceramic		PM10	PM2.5
	Foundries		PM10	PM2.5
Natural dust	Marine aerosol		PM10	PM2.5
	Volcanic dust		PM10	PM50
	Brake dust	PM10	TSP	PM2.5
	Deicing salt		PM10	
	Diesel		PM10	
Road dust	Exhaust		PM10	PM2.5
	Fuel oil burning	PM10	TSP	PM2.5
	Gasoline exhaust		PM10	
	Road dust	PM10	TSP	PM2.5
	Traffic	PM10	SP	PM2.5
	Petrochemical		PM10	PM2.5
	Power plant	PM10	TSP	PM2.5

**Table 4:** Worldwide LCPMS manufacturers, the number of different PM sensor models they offer, and the number of scientific papers that have investigated their performance, either in laboratory conditions or in the field.

Manufacturer	Number of Sensor Models	References in This Review	Manufacturer	Number of Sensor Models	References in This Review	Manufacturer	Number of Sensor Models	References in this Review
Alphasense	3	12	Honeywell	3	3	Sharp	2	10
Amphenol Advanced Sensors	6	NA	NanoSense	1	NA	Shinyei	5	3
Bjhike	1	NA	Inovafitness	2	3	Tianjin Figaro-isweek	1	NA
Cubic Sensor and Instrument Co, Ltd.	11	NA	Panasonic	2	NA	Winsen	3	2
EcologicSense	1	NA	Plantower	3	9	Yaguchi Electr. Corp.	1	NA
Elitech	1	NA	Samyoung S&C	2	1			
Grove Studio	1	NA	Sensirion	1	1			

**Table 6:** Technical characteristics. The first column reports, together with the manufacturer’s name, the device model, and the numbers related to the papers that discuss applications based on that sensor model. Column 2 refers to the LCPMS dimension and weight. Column 3 is related to the power supply required by the device and indicates the power input required to properly supply the sensor. In column 4, the maximum absorption of current during normal device operations is reported. In column 5, the current in mA consumed by the sensor when in sleep mode (if applicable) is shown. Column 6 reports the ability to regulate the power of the laser source, providing a chance for the designer to regulate the power consumption of the entire system. Column 7 reports the “Response/warm up time” for the various devices listed. This parameter indicates the minimum time required by the sensor to give a response after a query. Column 8 reports information related to the “Output interface (Bus type)/Level”. Column 9 reports the “Flow type/inlet-outlet position” parameter, which indicates how sampled air is able to enter into the detection chamber. Column 10, “Lifetime/ageing phenomena”, reports data related to the device’s lifetime reliability to provide information related to system maintenance. The last column, “Approximate Cost range”, shows the price range (synthetic parameter) at the time of this review (approximately low ≤ 15 EUR; 15 EUR < mid < 60 EUR; high ≥ 60 EUR) to outline, along with the other parameters, the quality/price ratio of each sensor.

Manufacturer/Model/Ref	Dimension (mm) and Weight(g)	Power Supply (V)	Working Current (mA)	Sleep Current (mA)/Low Power Operating Modalities	Laser Power Regulation	Response/Warm up Time (s)	Output Interface s/Level	Flux Type/Inlet-Outlet Position	Lifetime/Ageing Phenomena	Approximate Cost Range
Alphasense/OPC/N2/ [81,82,93,94,97,98,114,126,127,129]	64 × 75 × 60 /105	4.8–5.2	175	95 mA/laser at minimum power; fan off	Yes	1.4/10	SPI/-	FAN/opposite sides	NA	High
Honeywell/HPMA115S0- XXX/ [67,122,129,130]	43 × 3600 × 23.7/-	5	80	20 mA	No	6/-	UART/-	FAN	10y	Mid
Inovafitness/SDS011/ [57,67,82,113]	71 × 70 × 23/100	4.7–5.3	70	4 mA/laser and fan sleep/low power operating mode	Laser sleep	1/10	UART, PWM/3.3V	FAN/opposite side	Service life is up to 8000 h	Mid
Plantower/PMS 1003/ [80,131]	65 × 42 × 23/-	5–5.5	100	<1 mA/adaptative acquisition frequency	No	1–10/-	UART/3.3V	FAN/opposite side	MTTF ≥ 3 Year	Mid
Plantower/PMS 7003/ [55,81,82,124,129]	48 × 37 × 12/-	5–5.5	100	<1 mA/adaptative acquisition frequency	On/off	1–10/-	UART/3.3V	FAN/same side	MTTF ≥ 3 Year	Mid
Plantower/PMS A003/ [76,125]	35 × 38 × 12/-	5–5.5	100	<1 mA/adaptative acquisition frequency	No	1–10/-	UART/3.3V	FAN/same side	MTTF ≥ 3 Year	Mid
Sensirion/SPS30/ [118,132]	40.6 × 40.6 × 12.2/26	4.5–5.5	80	<50 μA/Sleep-Mode–Idle-Mode	NA	1/30	UART, I2C/-	FAN	>10 y/maximum long-term number concentration precision limit drift 20 to 1000 #/cm^3^ ± 12.5 #/cm^3^/year 1000 to 3000 #/cm^3^ ± 1.25% m.v./year	High
Sharp/GP2Y1010AU0F/ [63,65,67,83,134,135,136]	46 × 30 × 17.6/16	5	40	No	EXT	0.001/-	Analog/-	No/opposite side	Laser diode: 50% degradation/5 years	Low
Shinyei/PMS1/ [98,137]	71.4 × 76.4 × 36.7/130	12	380	NA	NA	NA	Ethernet/-	Heater	NA	NA
Shinyei/PPD20V/ [138,139]	88 × 60 × 20/38	5	160	NA	No	-/60	PWM/-	Heater	7y	NA
Shinyei/PPD42NJ/ [63,138,140]	59 × 45 × 22/24	5	90	NA	No	-/60	PWM/-	Auto suction by a built-in heater resistor	7y	NA
Shinyei/PPD60PV-T2/ [138,141]	88 × 60 × 22/-	5	140	NA	No	-/60	PWM/-	Heater	3y	NA
Winsen/ZH03/ZH03A/ZH03B/ [67,82,142]	50 × 32.4 × 21/-	5	120	<10 mA	NA	-/45	PWM/-	FAN/opposite site	3y in the air	Mid

**Table 7:** LCPMS grouped according to the four application scenarios defined for the devices listed in Table 4.

Sensors	Output Flexibility	Integration Complexity	IoT	Applications that Require Advanced Metrological Properties
Alphasense OPC/N2 [114]	●	●		●
Alphasense OPC/N3 [116]	●	●		●
Alphasense OPC/R1 [115]	●	●		
Amphenol Telaire SM-PWM-01C [144]				
Amphenol Telaire SM-PWM-01S [145]			●	
Amphenol Telaire SM-UART-01D [151]		●		
Amphenol Telaire SM-UART-01L+ [152]				●
Amphenol Telaire SM-UART-04L [153]		●		●
Amphenol Telaire Telaire DSF Series [170]		●		●
bjhike HK-A5 [154]		●		
Cubic Sensor and Instrument Co,Ltd PM1003 [146]				●
Cubic Sensor and Instrument Co,Ltd PM1006K [143]			●	
Cubic Sensor and Instrument Co,Ltd PM2008 [119]	●	●		●
Cubic Sensor and Instrument Co,Ltd PM2008M [155]	●	●		●
Cubic Sensor and Instrument Co,Ltd PM2009 [120]	●	●		●
Cubic Sensor and Instrument Co,Ltd PM2012 [158]	●	●	●	●
Cubic Sensor and Instrument Co,Ltd PM2105M [157]	●	●		●
Cubic Sensor and Instrument Co,Ltd PM2107 [156]		●	●	●
Cubic Sensor and Instrument Co,Ltd PM3006T [160]			●	●
Cubic Sensor and Instrument Co,Ltd PM3015 [159]			●	●
Cubic Sensor and Instrument Co,Ltd PM5000 [161]				●
EcologicSense NEXT-PM [117]		●		●
Elitech PM-900M [171]		●		
Grove Studio Laser PM2.5 Sensor (HM3301) [162]		●		●
Honeywell HPMA115C0-003 [122]	●	●	●	
Honeywell HPMA115C0-004 [122]	●	●		
Honeywell HPMA115S0-XXX [122]		●	●	
NanoSense PM2036 [163]		●	●	●
Inovafitness SDS011 [113]		●	●	
Inovafitness SDS018 [164]		●	●	●
Panasonic LED Type PM2.5 Sensor [147]		●		
Panasonic SN-GCJA5 Laser Type PM Sensor [165]		●		
Plantower PMS 1003 [131]	●	●		●
Plantower PMS 7003 [124]	●	●		●
Plantower PMS A003 [125]	●	●		●
SAMYOUNG S&C PSML [148]				
SAMYOUNG S&C PSMU [148]				
Sensirion SPS30 [119]	●	●	●	●
Sharp GP2Y1010AU0F [136]				
Sharp DN7C3CA007 [166]				
Shinyei PM sensor [137]		●		
Shinyei PPD20V [139]				
Shinyei PPD42NJ [140]				
Shinyei PPD60PV-T2 [141]				
Shinyei PPD71 [149]				
tianjinFigaro-isweek TF-LP01 [167]		●		
Winsen ZPH01 [150]				
Winsen ZH03/ZH03A/ZH03B [142]		●	●	
Winsen ZH06-I [168]		●	●	
YaguchiElectric Corp. SDS021 [169]		●	●	

**Table 8:** The most meaningful LCPMS parameters. The columns report the quality parameters used to evaluate performance compared to a reference instrument, which is reported in the last column.

	Ref	Test Year	PM Class	Accuracy		Completeness	Detection Limit	Measurement Range	Precision	Reference Instrument
				*R* ^2^	Bias μg/m^3^		μg/m^3^	μg/m^3^	%	
				* Tested in a laboratory setup						
Plantower PMS A003	[76]	2018	2.5	0.91				0–49	12	PDR-1200
Plantower PMS 1003	[80]	2019	10	0.91						Gravimetric FRM
Plantower PMS 5003	[80]	2019	10	0.7						Gravimetric FRM
Plantower PMS 1003	[80]	2019	2.5	0.88	56.9%					Gravimetric FRM
Plantower PMS 5003	[80]	2019	2.5	0.89	11.6%					PartisolTM 2025i Sequential Air Sampler)
Plantower PMS 7003	[55]	2019	2.5	0.96				16–75	16	TEOM SEMC/GRIMM 1.108
Plantower PMS 7003	[55]	2019	10	0.97				16–75	14	TEOM SEMC/GRIMM 1.109
Novasense SDS011	[57]	2018	2.5	0.96				3–79		TSI DustTrak DRX
Novasense SDS012	[57]	2018	10	0.91				3–90		TSI DustTrak DRX
Alphasense OPCN2	[93]	2018	2.5	0.9				0–300		Grimm1.108
Alphasense OPCN2	[93]	2018	10	0.84				0–350		Grimm1.108
Alphasense OPCN2	[129]	2019	2.5	0.50				0–35		TEOM AURN
Honeywell HPMA115S0	[129]	2019	2.5	0.77				0–35		TEOM AURN
Plantower PMS 5003	[129]	2019	2.5	0.76				0–35		TEOM AURN
Plantower PMS 7003	[129]	2019	2.5	0.73				0–35		TEOM AURN
ZH03A (Winsen)	[82]	2018	2.5	0.81	3.27			0–120	25	TEOM 1400a
Alphasense OPCN2	[82]	2018	2.5	0.61	8.36			0–120	37	TEOM 1400a
Plantower PMS 7003	[82]	2018	2.5	0.89	3.36			0–120	11	TEOM 1400a
Novasense SDS011	[82]	2018	2.5	0.9	4.76			0–120	12	TEOM 1400a
Alphasense OPCN2	[98]	2018	2.5	0.2		82.00%				grimm edm 180
Alphasense OPCN2	[98]	2018	10	0.46		82.00%				grimm edm 18
Shinyei PMS-SYS-1	[98]	2018	2.5	0.52		92.00%				grimm edm 180
Alphasense OPCN2	[126]	2017	10	0.81	0.32			0–250		Bam 1020
Alphasense OPCN2	[126]	2017	10	0.84	2.83					grimm11R
Alphasense OPCN2	[126]	2017	2.5	0.43	1.92					grimm11R
Alphasense OPCN2	[81]	2019	2.5	0.45				0–100		TEOM AURN
Plantower PMS 5003	[81]	2019	2.5	0.7				0–100		TEOM AURN
Plantower PMS 7003	[81]	2019	2.5	0.77				0–100		TEOM AURN
Alphasense OPCN2	[97]	2019	2.5	0.81	0–146					teom
Honeywell HPMA115S0	[130]	2019	2.5	0.58				0–72.9		grimm edm 180
Honeywell HPMA115S0	[67]	2019	2.5	0.99						TSI-3025A
Novasense SDS011	[67]	2019	2.5	0.90 *						TSI-3025A
ZH03A (Winsen)	[67]	2019	2.5	0.98						TSI-3025A
sharp GP2y	[67]	2019	2.5	0.96						TSI-3025A
Alphasense OPCN2	[94]	2018	2.5	0.78				0–70		Palas Fidas 200
PPD42NS	[138]	2018	2.5	0.8			9.1	0–500		TSI DustTrak
PPD20V	[138]	2018	2.5	0.98			4.6	0–500		TSI DustTrak
PPD60PV	[138]	2018	2.5	0.87			29	0–500		TSI DustTrak
sharp GPD2y1010AU0F	[134]	2018	2.5	0.99				0–8000		TSI AM510 ‘Sidepak’
sharp GPD2y1010AU0F	[135]	2017	2.5	0.99 *			10.93	0–1000		Alphasense OPC-N2
sharp GPD2y1010AU0F	[63]	2015	2.5	0.99 *			26.9	0–5000		TSI AM510 ‘Sidepak’
Shinyei PPD42NS,	[63]	2015	2.5	0.95			6.44	0–300		TSI SidePak
Samyoung DSM501A	[63]	2015	2.5	0.98			11.4	0–300		TSI SidePak
sharp GPD2y1010AU0	[136]	2012	10	0.99				0–3000		TSI AM510 ‘Sidepak’
sharp GPD2y1010AU0F	[83]	2015	2.5	0.98 *				0–140		Dusttrak 8520
sharp GPD2y1010AU0F	[83]	2015	10	0.91 *				0–120		Dusttrak 8520
sharp GPD2y1010AU0F	[65]	2016	2.5	0.95				30–6300	<6%	SMPS/CPC(GRIMM)-APS 3321
Sensirion SPS30	[132]	2019	2.5	0.83 *				?	?%	Grimm1.108
Alphasense OPC-N2	[127]	2016	2.5	0.99 *				10–10,000	4.2–16%	SMPS/CPC(GRIMM)-APS 3321

## Supplementary Materials

The following are available online at https://www.mdpi.com/1424-8220/20/23/6819/s1, Table S1: Supporting information for PM sensors Metrological characteristics

## Abbreviations

AQ	Air quality
DQO	Data quality objective
LCPMS	Low-cost PM (PM1, PM2.5, PM10) sensors
MMAD	Mass median aerodynamic diameter
MNB	Mean normalized bias
MNE	Mean normalized error
MLP	Multilayer perceptron
OPCs	Optical particle counters
PM	Particulate matters
RSs	Regulatory stations
RH	Relative humidity
TSP	Total suspended particles
HVAC	Heating ventilation air conditioning
IoT	Internet of Things
