# Correction: Alfano et al. A Review of Low-Cost Particulate Matter Sensors from the Developers’ Perspectives. *Sensors* 2020, *20*, 6819

**DOI:** 10.3390/s21093060

**Published:** 2021-04-28

**Authors:** Brigida Alfano, Luigi Barretta, Antonio Del Giudice, Saverio De Vito, Girolamo Di Francia, Elena Esposito, Fabrizio Formisano, Ettore Massera, Maria Lucia Miglietta, Tiziana Polichetti

**Affiliations:** 1 ENEA CR-Portici, TERIN-FSD Department, P.le E. Fermi 1, 80055 Portici, Italy; brigida.alfano@enea.it (B.A.); antonio.delgiudice@enea.it (A.D.G.); girolamo.difrancia@enea.it (G.D.F.); elena.esposito@enea.it (E.E.); fabrizio.formisano@enea.it (F.F.); ettore.massera@enea.it (E.M.); mara.miglietta@enea.it (M.L.M.); tiziana.polichetti@enea.it (T.P.); 2 Department of Physics, University of Naples Federico II, via Cinthia, 80100 Napoli, Italy; luigi.barretta3@unina.it; 3 STmicroelectronics, via R. De Feo, Arzano, 80022 Napoli, Italy

The authors wish to make the following corrections to this paper [1]:

In the original article, the sorting order of the reference list starting from reference no. 110 up to reference no. 172 was incorrect. The correct sorting order is reported below in correctly sorted reference list. 

Furthermore, reference no. 173 was missing and was wrongly referenced as no. 172. This was corrected adding reference no. 173:

173. Jayaratne, R.; Liu, X.; Ahn, K.H.; Asumadu-Sakyi, A.; Fisher, G.; Gao, J.; Mabon, A.; Mazaheri, M.; Mullins, B.; Nyaku, M.; et al. Low-cost PM2.5 Sensors: An Assessment of their Suitability for Various Applications. *Aerosol Air Q. Res.* **2020**, *20*, 520–532.

Additionally, the following errors to the citation numbering in the text were present:

In Table 5, sixth row: the reference number [76] was incorrectly written as [77].

Page 37, first row: the reference number [136] was incorrectly written as [137].

Table 7, 38th row: the reference number [118] was incorrectly written as [119].

Finally, in SI, the following corrections to the citation numbering apply:

Table S1 4th row: [153] was incorrectly written as [145].

Table S1 8th row: [153] was incorrectly written as [145].

Table S1 Row 41: both references were incorrect and were changed in [98], [137].

Table S2 Row 40: both references were incorrect and were changed in [98], [137].

The authors apologize for any inconvenience caused. We state that the scientific conclusions are completely unaffected. The original article has been updated accordingly.

Correctly sorted reference list (from no. 110 on)

110. Zaidan, M.A.; Motlagh, N.H.; Fung, P.L.; Lu, D.; Timonen, H.; Kuula, J.; Niemi, J.V.; Tarkoma, S.; Petaja, T.; Kulmala, M.; et al. Intelligent Calibration and Virtual Sensing for Integrated Low-Cost Air Quality Sensors. *IEEE Sens. J.* **2020**, *20*, 13638–13652.
111. Si, M.; Xiong, Y.; Du, S.; Du, K. Evaluation and calibration of a low-cost particle sensor in ambient conditions using machine-learning methods. *Atmos. Meas. Tech.* **2020**, *13*, 1693–1707.
112. Wang, W.V.; Lung, S.C.; Liu, C. Application of Machine Learning for the in-Field Correction of a PM2.5 Low-Cost Sensor Network. *Sensors* **2020**, *20*, 5002. Available online: https://www.mdpi.com/1424-8220/20/17/5002 (accessed on 21 November 2020).
113. Nova Sensor SDS011 Sensor Specification. Available online: https://www-sd-nf.oss-cn-beijing.aliyuncs.com/%E5%AE%98%E7%BD%91%E4%B8%8B%E8%BD%BD/SDS011%20laser%20PM2.5%20sensor%20specification-V1.4.pdf (accessed on 1 October 2019).
114. Alphasense OPC-N2 Sensor Specification. Available online: https://stg-uneplive.unep.org/media/aqm_document_v1/Blue%20Print/Components/Microcomputer%20and%20sensors/B.%20Dust%20Sensor%20Specifications/B.1%20Alphasense%20OPC%20N1/OPC-N2.pdf (accessed on 1 October 2019).
115. Alphasense OPC-R1 Sensor Specification. Available online: http://www.alphasense.com/WEB1213/wpcontent/uploads/2019/08/OPC-R1.pdf (accessed on 1 October 2019).
116. Alphasense OPC-N3 Sensor Specification. Available online: http://www.alphasense.com/WEB1213/wpcontent/uploads/2019/03/OPC-N3.pdf (accessed on 1 October 2019).
117. Available online: https://tera-sensor.com/technology/ (accessed on 1 October 2020).
118. Sensirion SPS30 Sensor Specification. Available online: https://www.sensirion.com/en/download-center/particulate-matter-sensors-pm/particulate-matter-sensor-sps30/ (accessed on 1 October 2019).
119. Cubic. Laser Particle Sensor PM2008. Available online: http://en.gassensor.com.cn/ParticulateMatterSensor/indoor/19/7/194/ (accessed on 1 October 2019).
120. Cubic. Dust Sensor PM2009. Available online: http://en.gassensor.com.cn/ParticulateMatterSensor/indoor/19/7/196/ (accessed on 1 October 2019).
121. Cubic. Outdoor Particulate Matter Measurement Technology. Available online: http://en.gassensor.com.cn/uploadfiles/2020/03/20200302112209222.pdf (accessed on 1 October 2019).
122. Honeywell. HPM Series Particulate Matter Sensors. Available online: https://sensing.honeywell.com/honeywell-sensing-particulate-hpm-series-datasheet-32322550 (accessed on 1 October 2019).
123. Cubic. Dust Sensor PM3006T. Available online: http://en.gassensor.com.cn/ParticulateMatterSensor/outdoor/19/9/208/ (accessed on 1 October 2019).
124. Plantower PMS7003 Sensor Specification. Available online: https://download.kamami.com/p564008-p564008-PMS7003%20series%20data%20manua_English_V2.5.pdf (accessed on 21 November 2020).
125. Plantower PMSA003 Sensor Specification. Available online: https://datasheet.lcsc.com/szlcsc/1810311017_Beijing-Plantower-PMSA003-A_C132744.pdf (accessed on 21 November 2020).
126. Mukherjee, A.; Stanton, L.G.; Graham, A.R.; Roberts, P.T. Assessing the utility of low-cost particulate matter sensors over a 12-week period in the Cuyama valley of California. *Sensors* **2017**, *17*, 1805.
127. Sousan, S.; Koehler, K.; Hallett, L.; Peters, T.M. Evaluation of the Alphasense optical particle counter (OPC-N2) and the Grimm portable aerosol spectrometer (PAS-1.108). *Aerosol Sci. Technol.* **2016**, *50*, 1352–1365.
128. Available online: http://www.alphasense.com/WEB1213/wp-content/uploads/2018/12/AAN-701-01.pdf (accessed on 29 November 2020).
129. Johnston, S.J.; Basford, P.J.; Bulot, F.M.; Apetroaie-Cristea, M.; Easton, N.H.; Davenport, C.; Foster, G.L.; Loxham, M.; Morris, A.K.; Cox, S.J. City scale particulate matter monitoring using LoRaWAN based air quality IoT devices. *Sensors* **2019**, *19*, 209.
130. Báthory, C.; Kiss, M.L.; Trohák, A.; Dobó, Z.; Palotás, Á.B. Preliminary research for low-cost particulatematter sensor network in E3S Web of Conferences. *EDP Sci.* **2019**, *100*, 00004.
131. Plantower PMS1003 Sensor Specification. Available online: http://www.aqmd.gov/docs/default-source/aqspec/resources-page/plantower-pms1003-manual_v2-5.pdf (accessed on 21 November 2020).
132. Kuula, J.; Mäkelä, T.; Aurela, M.; Teinilä, K.; Varjonen, S.; Gonzales, O.; Timonen, H. Laboratory evaluation of particle size-selectivity of optical low-cost particulate matter sensors. *Atmos. Meas. Tech. Dis.* **2019**, 1–21.
133. Li, J.; Li, H.; Ma, Y.; Wang, Y.; Abokifa, A.A.; Lu, C.; Biswas, P. Spatiotemporal distribution of indoor particulate matter concentration with a low-cost sensor network. *Build. Environ.* **2018**, *127*, 138–147.
134. Marinov, M.B.; Hensel, S.; Ganev, B.; Nikolov, G. Performance evaluation of low-cost particulate matter sensors. In Proceedings of the 2017 XXVI International Scientific Conference Electronics (ET), Sozopol, Bulgaria, 1–4 September 2017.
135. Olivares, G.; Longley, I.; Coulson, G. *Development of a Low-Cost Device for Observing Indoor Particle Levels Associated with Source Activities in the Home*; International Society of Exposure Science (ISES): Seattle, WA, USA, 2012.
136. Sharp GP2Y1010AU0F Sensor Specification. Available online: https://www.sharpsde.com/products/optoelectronic-components/model/GP2Y1010AU0F/#productview (accessed on 21 November 2020).
137. Shinyei University. PM Sensor PMS1. Available online: https://www.shinyei.co.jp/stc/eng/optical/main_pm2.html (accessed on 21 November 2020).
138. Johnson, K.K.; Bergin, M.H.; Russell, A.G.; Hagler, G.S. Field test of several low-cost particulate matter sensors in high and low concentration urban environments. *Aerosol Air Q. Res.* **2018**, *18*, 565–578.
139. Shinyei Kaisen PPD20V. Product Specification. Available online: http://c1170156.r56.cf3.rackcdn.com/UK_SHN_PPD20V_DS.pdf (accessed on 21 November 2020).
140. Shinyei PPD42NJ Sensor Specification. Available online: http://www.gvzcomp.it/index.php/it/shinyei?format=raw&task=download&fid=461 (accessed on 21 November 2020).
141. Shinyei PPD60PV Sensor Specification. Available online: http://www.gvzcomp.it/index.php/en/shinyei?format=raw&task=download&fid=463 (accessed on 21 November 2020).
142. Winsen. Laser Dust Module. Available online: https://www.winsen-sensor.com/d/files/air-quality/zh03-series-laser-dust-module-v2_0.pdf (accessed on 21 November 2020).
143. Cubic. LED Particle Sensor PM1006K. Available online: http://en.gassensor.com.cn/ParticulateMatterSensor/indoor/19/7/206/ (accessed on 1 October 2019).
144. Telaire. Smart Dust Sensor SM-PWM-01c. Available online: https://amphenol-sensors.com/en/component/edocman/225-sm-pwm-01c-application-note/download?Itemid=8248%20%27 (accessed on 21 November 2020).
145. Telaire. Smart Dust Sensor SM-PWM-01s. Available online: https://amphenol-sensors.com/en/component/edocman/478-telaire-sm-pwm-01s-smart-dust-sensor-datasheet/download?Itemid=8488 (accessed on 21 November 2020).
146. Cubic. LED Particle Sensor PM1003. Available online: http://en.gassensor.com.cn/ParticulateMatterSensor/indoor/19/7/205/ (accessed on 1 October 2019).
147. Panasonic. LED Type PM2.5 Sensor Specification. Available online: https://industrial.panasonic.com/ww/products/sensors/built-in-sensors/dust-sensor/pm25_led (accessed on 21 November 2020).
148. Samyoung S&G. PM2.5 Sensor. Available online: http://samyoungsnc.com/particle-sensor/ (accessed on 1 October 2019).
149. Shinyei Technology. Particle Sensor Unit PPD71. Available online: https://www.shinyei.co.jp/stc/eng/optical/main_ppd71.html (accessed on 21 November 2020).
150. Winsen ZPH01 Sensor Specification. Available online: https://www.winsen-sensor.com/d/files/PDF/Gas%20Sensor%20Module/PM2.5%20Detection%20Module/ZPH01%20Particles%20Sensor%20Module%20V1.0.pdf (accessed on 21 November 2020).
151. Telaire. SM-UART-01D Dual Channel Dust Sensor. Available online: https://amphenol-sensors.com/en/component/edocman/477-telaire-sm-uart-01d-dual-channel-dust-sensor-datasheet/download?Itemid=8488 (accessed on 21 November 2020).
152. Telaire. SM-UART-01L+ Laser Dust Sensor PM2.5. Available online: https://amphenol-sensors.com/en/component/edocman/429-telaire-sm-uart-01l-laser-dust-sensor-datasheet/download?Itemid=8248%20%27 (accessed on 21 November 2020).
153. Amphenol SM UART 04l Sensor Specification. Available online: https://amphenol-sensors.com/en/component/edocman/514-telaire-sm-uart-04l-laser-dust-sensor-application-note/download?Itemid=8488%20%27 (accessed on 21 November 2020).
154. HK-A5 Laser PM2.5/10 Sensor. Available online: https://github.com/Arduinolibrary/DFRobot_PM2.5_Sensor_module/raw/master/HK-A5%20Laser%20PM2.5%20Sensor%20V1.0.pdf (accessed on 21 November 2020).
155. Cubic. Dust Sensor PM2008M-M. Available online: http://en.gassensor.com.cn/ParticulateMatterSensor/indoor/19/7/195/ (accessed on 1 October 2019).
156. Cubic. Laser Particle Sensor PM2107. Available online: http://en.gassensor.com.cn/ParticulateMatterSensor/indoor/19/7/193/ (accessed on 1 October 2019).
157. Cubic. Laser Particle Sensor PM2105M. Available online: http://en.gassensor.com.cn/ParticulateMatterSensor/indoor/19/7/192/ (accessed on 1 October 2019).
158. Cubic. Laser Particle Sensor PM2012. Available online: http://en.gassensor.com.cn/ParticulateMatterSensor/indoor/19/7/198/ (accessed on 1 October 2019).
159. Cubic PM3015. Available online: https://drive.google.com/file/d/1fp7BszNmv6NWFFmpkmHPxV8PQcIRfQK8/view (accessed on 1 October 2019).
160. Cubic. Dust Sensor PM3006T. Available online: http://en.gassensor.com.cn/uploadfiles/2020/07/20200702111723790.pdf (accessed on 1 October 2019).
161. Cubic. Particle Counter PM5000. Available online: http://en.gassensor.com.cn/ParticulateMatterSensor/indoor/19/7/202/ (accessed on 1 October 2019).
162. Seeed The Lot Hardware Unabler. Grove—Laser PM2.5 Sensor (HM3301). Available online: https://wiki.seeedstudio.com/Grove-Laser_PM2.5_Sensor-HM3301/ (accessed on 21 November 2020).
163. Nanosense.PM2036 Sensor Specification. Available online: http://nano-sense.com/wp-content/uploads/2018/09/PM2036NS-Datasheet-NanoSense-V4.2-20171006.pdf (accessed on 21 November 2020).
164. Nova Fitness SDS018 Sensor Specification. Available online: https://www-sd-nf.oss-cn-beijing.aliyuncs.com/%E5%AE%98%E7%BD%91%E4%B8%8B%E8%BD%BD/SDS018%20Laser%20PM2.5%20Product%20Spec%20V1.5.pdf (accessed on 21 November 2020).
165. Panasonic.laser type PM Sensor. Available online: https://industrial.panasonic.com/ww/products/sensors/built-in-sensors/dust-sensor/pm_laser (accessed on 21 November 2020).
166. Sharp DN7C3CA007 Sensor Specification. Available online: https://www.sharpsde.com/products/optoelectronic-components/model/DN7C3CA007/ (accessed on 1 October 2019).
167. Isweek TF-LP01 Sensor Specification. Available online: https://www.isweek.com/Uploads/20180604/5b14bb38b82aa.pdf (accessed on 21 November 2020).
168. Winsen ZH06 Sensor Specification. Available online: https://www.winsen-sensor.com/sensors/dust-sensor/245.html (accessed on 21 November 2020).
169. Yaguchi Electric Pocket PM2.5 Sensor Specification. Available online: https://cdn.sparkfun.com/assets/parts/1/2/2/7/5/Pocket_PM2.5_sensor_spec.pdf (accessed on 21 November 2020).
170. Amphenol Telair Sensor Specification. Available online: https://www.amphenol-sensors.com/en/component/edocman/559-telaire-dsf-series-automotive-pm2-5-in-cabin-sensor-product-datasheet/download?Itemid=8488 (accessed on 21 November 2020).
171. Elitech PM900M Sensor Specification. Available online: https://www.elitecheu.com/collections/temtop-euparticle-counter/products/temtop-pm-900m-laser-particle-sensor-for-particulate-matter-pm1-0-pm2-5-pm10 (accessed on 21 November 2020).
172. Han, I.; Symanski, E.; Stock, T.H. Feasibility of using low-cost portable particle monitors for measurement of fine and coarse particulate matter in urban ambient air. *J. Air Waste Manag. Assoc.* **2017**, *67*, 330–340.
173. Jayaratne, R.; Liu, X.; Ahn, K.H.; Asumadu-Sakyi, A.; Fisher, G.; Gao, J.; Mabon, A.; Mazaheri, M.; Mullins, B.; Nyaku, M.; et al. Low-cost PM2.5 Sensors: An Assessment of their Suitability for Various Applications. *Aerosol Air Q. Res.* **2020**, *20*, 520–532.
