# Supplementary material for: A Review of Low-Cost Particulate Matter Sensors from the Developers’ Perspectives
Source: Sensors (Basel). 2020 Nov 29;20(23):6819. doi: 10.3390/s20236819 (PMC7730878; doi:10.3390/s20236819)
Supplement: Supplementary file 1 [file sensors-20-06819-s001.pdf]

Table S1: Supporting information for PM sensors Metrological characteristics

The metrological characteristics obtained from the official manufacturers' websites and freely available technical sheets. The first column refers to the manufacturers' name, the device model, and the papers and refers to applications based on that sensor model. Column two reports the device's basic operating mechanism and its main application scenario. Column three reports the device output in µg/m³ or pcs/l. Columns four, five, and six report, respectively, the measurement range and its resolution in units corresponding to the device output, as well as the indication error related to the device output. Columns seven and eight are, respectively, the sensor operating temperature range and the RH operating range, respectively. Column nine reports the minimum detectable value of the particle size for each of the sensors discussed. Column ten reports information on device calibration and the way it was performed. In column eleven, the possibility to access raw data is shown.

**Manufacturer/ Model/ Ref**	**Type/Main application**	**Main measured output data**	**Measurement range (µg/m³)**	**Concentration resolution (µg/m³)**	**Working temperature range (°C)**	**Working humidity range (%RH noncondensing)**	**Error**	**Particle diameter resolution or range(µm)**	**Manufacturer Calibration [or other laboratory tests]**	**Raw data availability**
Alphasense/ OPC/N2/ [81] [82] [93] [94] [97] [98] [114] [126] [127] [129]	particulate monitor/outdoor	PM1, PM2.5 and PM10	1000 (particles/second)	0.01	-20 to +50	0-95%	NA	0.38 - 17	Method defined by European Standard EN 481/ TSI3330-GRIMM1.108 comparison	16 bins/1.4 to 10µm/modifiable particle density value
Alphasense/ OPC/N3 [116]	particulate monitor/indoor/outdoor/recommend in very dusty environments.	PM1, PM2.5, PM10	0-2000	0.01	-10 to +50	0-95%	NA	0.35-40	TSI OPS 3330; Grimm 1.108 instruments comparison	0.35-40 24 bins

Alphasense/ OPC/R1 [115]	particulate monitor/indoor-outdoor	PM1, PM2.5, PM10	10000 (particles/ second)	NA	-10 to +50	0-95%	NA	0.35 - 12.4	Comparison of PM2.5/PM10 monitoring by OPCR1 sensor and TSI OPS 3330 and DustTrak instruments(ambient air of a work shop)/calibrated using Polystyrene Spherical Latex Particles (PSLs) of a known diameter and known RI	0.35 to 12.4;16 bins; allows a different value to be set for each size bin to correct for particle density variation with particle size
Amphenol Advanced Sensors/ SM-PWM-01C [144]	dust sensor/indoor	PM2.5, PM10	0.01-3000	noise lim	-10 to +45	0-85%	On calibration graph	1	Cigarette smoke	2xPWM scattered signal(PM:1-2µm;PM:3-10µm
Amphenol Advanced Sensors/ Telaire SM-PWM-01S [145]	SMART dust sensor/ indoor-outdoor	1-3µm and 3-10µm	NA	NA	-10 to +45	0-85%	NA	1 - 20	cigarette smoke	Yes- 2xPWM scattered signal(PM:1-2µm;PM:3-10µm
Amphenol Advanced Sensors/ Telaire SM-UART-01D [151]	dual channel dust sensor/ indoor-outdoor; automotive;harsh environment	NA	NA	NA	-10 to 70	0-95%	NA	NA	NA	Yes
Amphenol Advanced Sensors/ Telaire SM-UART-01L+ [152]	PM2.5 laser dust sensor/-	PM10	1-999	1	-10 to +50	35-85%	0-100±15µ g/m³; 101-999µ g/m³±15%	0.3	NA	NA

Amphenol Advanced Sensors/ Telaire SM-UART-04L [145]	PM2.5-10 particulate dust sensor/-	PM2.5, PM10	1-999	1	-10 to +50	0-95%	0-100±10μ g/m^3;<br>101-999μ g/m^3±10%	0.3	NA	NA
Amphenol Advanced Sensors/ Telaire Telaire DSF Series [170]	/<br>indoor-outomotive	PM2.5	0 to 500	1	-40 to +80	NA	0-100±15μg/m^3;<br>100-500μg/m^3±15%	NA	Calibrated KCI	Temperature, PM2.5
Bjhike/ HK-A5 [154]	particle sensor/indoor	PM2.5/10	0-999	NA	-20 to +50	0-80%	NA	0.3 - 10	NA	NA
Cubic Sensor and Instrument Co,Ltd/ PM1003 [146]	IR LED particle sensor/indoor	PM2.5	0-500	NA	-10 to +50	0-95%	0-100±30μg/m^3; 100-500 μg/m^3±30% (25±2℃, 50±10%RH)	1.0 - 10	NA	No
Cubic Sensor and Instrument Co,Ltd/ PM1006K [143]	LED particle sensor/indoor	PM1.0, PM2.5, PM10	0-1000	1	-20 to +75	0-95%	Maximum between ±20μg/m^3 and ±20% (@voltage 5.0V, 25±2℃, 50±10%RH)	0.3 - 10	TSI-8530, cigarette smoke	No
Cubic Sensor and Instrument Co,Ltd/ PM2008 [119]	laser particle sensor/indoor	PM1.0, PM2.5, PM10	0~5000	1	-10 to +60	0-95%	PM1.0&PM2.5: 0-35±10μg/m^3 >35μg/m^3±10%;<br>Condition: 25±2℃,50±10%RH; PM10: 0 - 100±25μg/m^3 101 - 1000μg/m^3, ±25%<br>Condition: 25±2℃,50±10%RH,	0.3 - 10	Reference instrument: GRIMM<br>Dust Source: Cigarette +Arizona A1	6 bins particle number; Grimm mass concentration/<br>TSI mass concentration/Setup particle calibrated coefficient

Cubic Sensor and Instrument Co,Ltd/ PM2008M [155]	laser particle sensor/indoor	PM1.0, PM2.5, PM10	0-1000	1	-10 to +60	0-95%	PM1.0&PM2.5: 0 - 35±5μg/m³ ; 35-500μg/m³±15%; Condition: 25±2℃,50±10%RH; PM10: 0 - 100±30μg/m³ ; 101 - 500μg/m³±30%; Condition: 25±2℃,50±10%RH	0.3 - 10	Reference instrument: GRIMM Dust Source: Cigarette +Arizona A1	6 bins particle number; Grimm mass concentration/ TSI mass concentration/Setup particle calibrated coefficient
Cubic Sensor and Instrument Co,Ltd/ PM2009 [120]	laser particle sensor/indoor	PM1.0, PM2.5, PM10	0-5000	1	-20 to +60	0-95%	PM1.0&PM2.5: 0-50±5μg/m³ ; 50-100±10μg/m³ ;100-300μg/m³±15% Condition: 25±2℃,50±10%RH; PM10:0-100±25μg/m³; 100-300μg/m³±25% Condition: 25±2℃,50±10%RH	0.3 - 10	Reference instrument: GRIMM Dust Source: Cigarette +Arizona A1	6 bins particle number; Grimm mass concentration/ TSI mass concentration/Setup particle calibrated coefficient
Cubic Sensor and Instrument Co,Ltd/ PM2012 [158]	Laser Particle sensor/indoor	PM1.0 PM2.5 PM10	0-5000	1	10 to +60	0-95%	PM2.5/PM1.0: 0-100±10μg/m³ ; 101-500μg/m³±10% PM10: 0-100±25μg/m³ ; 101-500μg/m³±25% (GRIMM, 25±2℃, 50 ±10%RH)	0.3 - 10	(GRIMM, 25±2℃, 50 ±10%RH)	6 bins particle number; Grimm mass concentration/ TSI mass concentration/Setup Particle Calibrated Coefficient

Cubic Sensor and Instrument Co,Ltd/ PM2105M [157]	laser particle sensor/indoor	PM1.0, PM2.5, PM10	0-1000	1	-10 to +60	0-95%	PM1.0&PM2.5: 0 - 35±5μg/m³ ; >35μg/m³±15% Condition: 25±2℃, 50±10%RH; PM10: 0 -100±30μg/m³; 101 - 1000μg/m³±30% Condition: 25±2℃, 50±10%RH,	0.3 - 10	Reference instrument: GRIMM/TSI Dust source: Cigarette	6 binsParticle number;Grimm mass concentration/ TSI mass concentration/Set up particle calibrated coefficient
Cubic Sensor and Instrument Co,Ltd/ PM2107 [156]	laser Particle sensor/indoor	PM2.5	0-1000	1	-10 to +50	0-95%	0-35±5μg/m³; >35μg/m³±15% Condition: 25±2℃, 50±10%RH,	0.3 - 10	Reference instrument: TSI8530 Dust Source: Cigarette	No
Cubic Sensor and Instrument Co,Ltd/ PM3006T [160]	outdoor laser particle sensor module/outdoor	PM1.0, PM2.5, PM10	0-1000	NA	-30 to +70	0-95%	PM1.0/PM2.5: <35 ±5μg/m³; 35-100±10μg/m³ 100-1000μg/m³±10% Condition: 25±2℃,50±10%RH; PM10: ≤100±15μg/m³ 100-1000μg/m³±15% Condition: 25±2℃,50±10%RH,	0.3 - 10	Reference instrument: GRIMM	Setup particle calibrated coefficient of PM1.0, PM2.5, and PM10

Cubic Sensor and Instrument Co,Ltd/ PM3015 [159]	laser particle sensor/ indoor, outdoor	PM1.0, PM2.5, PM10	0 - 500	1	-25 to +60	0-95%	PM1.0&PM2.5: ≤ 100 ±10µg/m³<br>>100µg/m³ ±10%<br>(Reference instrument: GRIMM<br>Conditions: 25±2°C,50±10%RH)	NA	NA	Yes
Cubic Sensor and Instrument Co,Ltd/ PM5000 [161]	laser particle count sensor module/indoor,outdoor	6bins(number pcs/L):0.3µm, 0.5µm, 1.0µm, 2.5µm, 5.0µm, 10µm	0-10000000 (particles/l)	1 (pcs/l)	-30 - +70	0-95%	≤100±30pcs/L<br>> 100pcs/L±30%<br>Condition: 25±2℃,50±10%RH	0.3 - 10	Reference instrument: TSI9306	6 bins(number pcs/L):0.3µm, 0.5µm, 1.0µm2.5µm, 5.0µm, 10µm
EcologicSense/ NEXT-PM [117]	particulate matter OEM sensor/indoor/outdoor	PM1 - PM2,5 - PM10	0 - 1000	NA	-20 to +70	0-95%	Linearity error <5 %<br>Repeatability error <3 %	0.3 - 10	NA	Yes
Elitech/ PM-900M [171]	particle sensor/<br>indoor-automotive	PM1.0, PM2.5, PM10	effective (PM2.5): 0-500	1	-10 to +60	NA	0-100±10µg/m³;100-500 µg/m³±10%	0.3	NA	Yes
Grove Studio/ Laser PM2.5 Sensor (HM3301) [162]	/indoor	PM2.2, PM10, TSP	1-500 (effective range)<br>1-1000 (maximum range)	1	-10 to +60	10-90%	100-500µ g/m³±10%<br>0-100±10µ g/m³	0.3	NA	NA

Honeywell/ HPMA115C0-003 [122]	laser-based light scattering particle sensing/indoor - automotive	PM1.0, PM2.5, PM4.0, PM10	0-1000	1	-20 to +70	0-95%	PM2.5: 0-100±15μg/m³; 100-1000μg/m³±15 %; PM1.0, PM4.0, PM10: 0-100±25μg/m³; 100-1000μg/m³±25%;	NA	NA	Customer adjustment coefficient
Honeywell/ HPMA115C0-004 [122]	laser-based light scattering particle sensing/indoor	PM1.0, PM2.5, PM4.0, PM10	0-1000	1	-20 to +70	0-95 %	PM2.5: 0-100±15μg/m³; 100-1000μg/m³±15 %; PM1.0, PM4.0, PM10: 0-100±25μg/m³; 100-1000μg/m³±25%;	NA	NA	Customer Adjustment Coefficient
Honeywell/ HPMA115S0-XXX/ [67] [122] [129] [130]	laser-based light scattering particle sensing/ indoor-automotive	PM2.5 PM10	0-1000	1	-20 to +50	0-95%	PM2.5: 0 - 100 ±15 μg/m³ PM2.5: 100 - 1000 μg/m³ ±15 %	NA	NA	Customer adjustment coefficient
Inovafitness/ SDS011/ [57] [67] [82] [113]	laser based PM2.5;10 sensor/indoor	PM2.5,PM10	0-1000	0.3	-10 to +50	0-70%	Maximum between ±15% and ±10μg/m³	0.3-10	NA	No
Inovafitness/ SDS018 [164]	laser based PM2.5;10 sensor/indoor	PM2.5,PM10	0-1000	0.3	-10 to +50	0-70%	Maximum between ±15% and ±10μg/m³	0.3-10	NA	No
NanoSense/ PM2036 [163]	particle sensor/ indoor-outdoor	PM2.5, PM10	0 - 1000	NA	-20 to +50	0-95%	< 50 ± 10μg/m³ 50-100± 15μg/m³ > 100μg/m³ ± 15%	0.3	NA	Yes

Panasonic/ LED Type PM2.5 Sensor [147]	particle sensor/indoor	PM 2.5	0-300	NA	NA	NA	NA	0.5	NA	Yes
Panasonic/ SN-GCJA5<br>Laser Type PM Sensor [165]	particle sensor/indoor	PM2.5, PM10 & PM1, cigarette smoke, house dust	0 – 2000	NA	-10 to +60	0-95%	±10%	0.3	Particle<br>generator<br>Cigarette smoke suction machine<br>(complies with JEM1467)<br>Customized particle generator<br>Dust meter DUSTTRAK II Aerosol Monitor 8530<br>(with PM2.5 impactor)<br>DUSTTRAK II Aerosol Monitor 8530<br>(with PM10 impactor)	Yes
Plantower/ PMS 1003/ [80] [131]	laser based particle concentration sensor/indoor	PM 1, PM2.5, PM10	0-500	1	-10 to +60	0-99%	100-500 μg/m³ ±10%<br>0-100 ± 10 μg/m³	0.3	Standard Particles/<br>atmospheric environment	6 bins particle number; standard particles concentration/<br>atmospheric environment concentration

Plantower/ PMS 7003/ [55] [81] [82] [124] [129]	laser based particle concentration sensor/indoor	PM 1, PM2.5, PM10	0-500	1	-10 to +60	0-99%	100-500$\mu g/m^3$ ±10%<br>0-100 ± 10 $\mu g/m^3$	0.3	Standard Particles/<br>atmospheric environment	6 bins particle number; standard particles concentration/<br>atmospheric environment concentration
Plantower/ PMS A003/ [76] [125]	laser based particle concentration sensor/indoor	PM 1, PM2.5, PM10	0-500	1	-10 to +60	0-99%	100-500 $\mu g/m^3$ ± 10%<br>0-100 ± 10 $\mu g/m^3$	0.3	Standard Particles/<br>atmospheric environment	6 bins particle number; standard particles concentration/<br>atmospheric environment concentration
SAMYOUNG S&C/ PSML [148]	PM 2.5 sensor/<br>indoor-outdoor	PM2.5/PM1.0	0-500 (effective) 0-900 (maximum)	NA	-10 to +65	0-95%	100-500$\mu g/m^3$±25% ; 0-100±25$\mu g/m^3$	0.3	Particle Source : Cigarette<br>Reference Instrument : GRIMM 11-A	NA
SAMYOUNG S&C/ PSMU [148]	PM 2.5 sensor/indoor-outdoor	PM2.5/PM1.0	0-500 (effective) 0-900 (maximum)	NA	-10 to +65	0-95%	100-500$\mu g/m^3$±25% ; 0-100±25$\mu g/m^3$	0.3	Particle Source : Cigarette<br>Reference Instrument : GRIMM 11-A	NA
Sensirion/ SPS30/ [118] [132]	particulate matter sensor/<br>indoor-outdoor	PM1.0, PM2.5, PM4, PM10	0-1000	1	10 to +40	20–80%	PM1, PM2.5:<br>0 - 100 ± 10 $\mu g/m^3$<br>100 - 1000 $\mu g/m^3$ ± 10%<br>PM4, PM10:<br>0 - 100 ± 25 $\mu g/m^3$<br>100 - 1000 $\mu g/m^3$ ± 25%	0.3	PM2.5 mass concentration Calibrated to TSI DustTrak™<br>DRX 8533 Ambient Mode<br>PM2.5 number concentration Calibrated to TSI OPS 3330	5 bins particle number

Sharp/ DN7C3CA007 [166]	dust sensor with particle separator /-	PM2.5	25-500	noise lim	-10 to +60	10-90%	NA	NA	Hipresica(siO2)2.5 um<br>-	12..13 18..20 number concentration PM2.5 [#/cm³]
Sharp/ GP2Y1010AU0F/ [63] [65] [67] [83] [134] [135] [136]	LED based dust sensor/indoor	PM10	0-500	noise lim	-10 to +65	NA	NA	NA	Cigarette smoke reference : dust monitor (P-5L2: manufactured by SHIBATA SCIENTIFIC TECHNOLOGY LTD)	10..11 15..17 number concentration PM1.0 [#/cm³]
Shinyei/ PMS1/ [98] [128]	particulate sensor/-	PM 2.5	0 - 200	NA	-10 to +45	20-85%	NA	0.3	NA	NA
Shinyei/ PPD20V/ [138] [139]	/indoor	PM10(pcs/litre)	0-30000 (particles/liter)	NA	0 to +40	0-95%	NA	1	Cigarette smoke, concentration reference:Rion Kc01/<br>Drop test, Vibration, high temperature and humidity endurance	Yes
Shinyei/ PPD42NJ/ [63] [138] [140]	particle sensor unit/indoor	PM2.5, PM10	0 - 8000 (particles/283ml)	NA	0 to +45	0-95%	NA	1	Cigarette smoke, weight concentration reference: sibata LD5reference, concentration reference:Rion Kc01/<br>Drop test, Vibration, high temperature and humidity endurance,	16..17 24..26 number concentration PM10 [#/cm³]

Shinyei/ PPD60PV-T2/ [138] [141]	particulate sensor/indoor	PM10(pcs/litre)detects air borne particles from cleanliness class 100000 to 1000000	0 to 20000 (particles/283ml) (0.5um range particle)	NA	0 to +45	0-95%	NA	0.5	Cigarette smoke, concentration reference:Rion Kc01/ Drop test, Vibration, high temperature and humidity endurance	Yes
Shinyei/ PPD71 [149]	/indoor	NA	0- 500	NA	-10 to +60	0-95%	NA	0.5	Cigarette smoke	14..15 21..23 number concentration PM4.0 [#/cm³]
TianjinFigaro-isweek/ TF-LP01 [167]	laser particulate matter sensor/indoor	PM1.0 ,PM2.5 ,PM10	0-999	1	-10 to +50	0-95%	NA	NA	NA	Yes
Winsen/ ZH03/ZH03A/ZH03B/ [67] [82] [142]	particles sensor PM2.5 dust sensor/indoor	PM1.0, PM2.5, PM10	PM2.5: 0-1000	NA	-10 to +50	0-85%	NA	0.3	NA	NA
Winsen/ ZH06- I [168]	laser dust sensor/indoor-	PM1.0、PM2.5、PM10	NA	NA	- 10 to +60	0-80%	NA	0.3	NA	NA
Winsen/ ZPH01 [150]	particles sensor/indoor-	PM2.5	15000 (particles/283ml)	NA	0 to +50	0-90%	NA	1	NA	NA
YAGUCHI ELECTRIC CORP./ SDS021 [169]	particle sensor/indoor-outdoor	PM1, PM2.5, PM10	0-1000	NA	-20 to +60	0-70%	Maximum between ±15% and ±10μg/m³	0.3	NA	Yes

Table S2 : Supporting information for Technical characteristics

The first column reports, together with the manufacturers' name, the device model and the numbers related to the papers that discuss applications based on that sensor model. Column 2 refers to the LCPMS dimension and weight. Column 3 is related to the power supply required by the device and indicates the power input required to properly supply

the sensor. In column 4, the maximum absorption of current during normal device operations is reported. In column five, the current in mA consumed by the sensor when in sleep mode (if applicable) is shown. Column 6 reports the ability to regulate the power of the laser source, providing a chance for the designer to regulate the power consumption of the entire system. Column 7 reports the "Response/warm up time" for the various devices listed. This parameter indicates the minimum time required by the sensor to give a response after a query. Column 8 reports information related to the "Output interface (Bus type)/Level". Column 9 reports the "Flow type/inlet-outlet position" parameter, which indicates how sampled air is able to enter into the detection chamber. Column 10, "Lifetime/ageing phenomena", reports data related to the device's lifetime reliability to provide information related to system maintenance. The last column, "Approximate Cost range", shows the price range (synthetic parameter) at the time of this review (approximately low<=15 EUR; 15 EUR<mid<60EUR ; high>=60 EUR) to outline, along with the other parameters, the quality / price ratio of each sensor.

**Manufacturer/ Model/ Ref**	**Dimension (mm) and weight (g)**	**Power supply (V)**	**Working current (mA)**	**Sleep current (mA)/Low power operating modalities**	**Laser power regulation**	**Response/Warm up time (sec)**	**Output interface s/Level**	**Flux type/Inlet-outlet position**	**Lifetime/Ageing phenomena**	**Approximate cost range**
Alphasense/ OPC/N2/ [81] [82] [93] [94] [97] [98] [114] [126] [127] [129]	64x75x60/105	4.8 to 5.2	175	95mA/laser at minimum power; fan off	Yes	1.4/10	SPI/-	FAN/opposite sides	NA	High
Alphasense/ OPC/N3 [116]	75x63.5x60/105	4.8 to 5.2	180	<45mA	Yes	1-30/-	SPI/-	FAN/opposite sides	NA	NA
Alphasense/ OPC/R1 [115]	72x21.5x25.5	4.8 to 5.2	95	<5mA	Yes	1-30/-	SPI/-	FAN/different side	NA	High
Amphenol Advanced Sensors/ SM-PWM-01C [144]	59x46x18/20	5	90	NO	No	5-30/120	2x PWM/3.3V	Hot air (forced natural)/same side	NA	Low

Amphenol Advanced Sensors/ Telaire SM-PWM-01S [145]	46x34x17.6/10	5	60	NA	No	NA	PWM,UART/-	Auto suction by a built-in heater resistor	NA	Low
Amphenol Advanced Sensors/ Telaire SM-UART-01D [151]	x-x-x/-	12	80	NA	No	-/30	UART/-	FAN	NA	Mid
Amphenol Advanced Sensors/ Telaire SM-UART-01L+ [152]	43x45x18/-	5	100	NA	NA	15/-	UART/-	FAN	NA	Mid
Amphenol Advanced Sensors/ Telaire SM-UART-04L [145]	48x12x37	5	100	NA	NA	5/-	UART/-	FAN	NA	Mid
Amphenol Advanced Sensors/ Telaire Telaire DSF Series [170]	78x51x33/-	9 to 16	NA	NA	No	15/60	LIN/-	NA	NA	NA
Bjhike/ HK-A5 [154]	46×35×20 /-	5	120	<200μA	NA	10/-	UART/-	FAN	> 5y	Mid
Cubic Sensor and Instrument Co,Ltd/ PM1003 [146]	59x45x17.2/26	5	90	NA	No	30/-	PWM,UART/-	Auto suction by a built-in heater resistor/same side	NA	NA

Cubic Sensor and Instrument Co,Ltd/ PM1006K [143]	46.2x34.1x18/17.4	5	30	NA	No	8/-	UART, PWM/4.5V	NO FAN/opposite side	MTTF: 5y	NA
Cubic Sensor and Instrument Co,Ltd/ PM2008 [119]	48×40×12/-	5	100	≤200µA/low power acquisition mode	No	1/8	UART,PWM, I2C/3.3V,5V	FAN/same side	MTTF: 37297 hrs (continuous turn on)	Mid
Cubic Sensor and Instrument Co,Ltd/ PM2008M [155]	48×40×12/-	5	100	≤200µA/low power acquisition mode	No	1/8	UART,PWM, I2C/3.3V,5V	FAN/same side	MTTF: 37297 hrs (continuous turn on)	Mid
Cubic Sensor and Instrument Co,Ltd/ PM2009 [120]	50x38x21/-	5	100	< 20mA/low power acquisition mode	No	1/8	UART, I2C/3.3V,5V	FAN/same side	MTTF: 37297 hrs (continuous turn on)	Mid
Cubic Sensor and Instrument Co,Ltd/ PM2012 [158]	38x35x12/-	5	100	≤200µA/low power acquisition mode	No	1/8	UART,I2C/ 3.3V,5V)	FAN/Two Type: Same side/ opposite sides	MTTF 110000 hrs (continuous turn on)	Mid
Cubic Sensor and Instrument Co,Ltd/ PM2105M [157]	42×35×23.7/-	5	100	< 20mA/low power acquisition mode	No	1/8	UART, PWM I²C/3.3V,5V	FAN/opposite side	MTTF 37297 hrs (continuous turn on)	Mid
Cubic Sensor and Instrument Co,Ltd/ PM2107 [156]	58.7x45x13.9/-	5	100	< 20mA/Low Power Acquisition Mode	No	1/8	UART, PWM I²C/3.3V,5V	FAN/same side	MTTF: 16910 hrs (continuous turn on)	Mid

Cubic Sensor and Instrument Co,Ltd/ PM3006T [160]	85x74x24.9/-	5	250	<20mA/low power acquisition mode	No	1/8	UART,I2C/3.3V,5V)	FAN/opposite sides	Life span ＞3 y/laser diode MTTF>88000 hours	NA
Cubic Sensor and Instrument Co,Ltd/ PM3015 [159]	42×35×23.7/-	5	100	<20mA	No	1/8	UART,I2C/3.3V,5V	FAN	37297hr (continuous turn on)	NA
Cubic Sensor and Instrument Co,Ltd/ PM5000 [161]	85x74x24.9/-	5	250	<20mA	No	1/8	UART_TTL/I2C（3.3V/5V）	FAN/opposite sides	Life span ≥3y	NA
EcologicSense/ NEXT-PM [117]	62x52x23/-	5	250	1mA	No	NA	UART,MODBUS/-	FAN	(MTTF) : 10 000 hrs	NA
Elitech/ PM-900M [171]	NA	5	100	NA	No	NA	NA	FAN	MTTF 110000 hrs (continuous turn on)	Mid
Grove Studio/ Laser PM2.5 Sensor (HM3301) [162]	40×38×15/-	5	75	150µA	NA	-/30	UART,I2C /3.3V	FAN/different Side	2y min (indoor)	Mid
Honeywell/ HPMA115C0-003 [122]	44x36x12/-	5	80	20mA	No	6/-	UART/-	FAN	10y	Mid
Honeywell/ HPMA115C0-004 [122]	44x36x12/-	5	80	20mA	No	6/-	UART/-	FAN	10y	Mid
Honeywell/ HPMA115S0-XXX/	43x36,00x23,7/-	5	80	20mA	No	6/-	UART/-	FAN	10y	Mid

[67] [122] [129] [130]										
Inovafitness/ SDS011/ [57] [67] [82] [113]	71x70x23/100	4.7 to 5.3	70	4mA/laser and fan sleep/low power operating mode	Laser sleep	1/10	UART,PWM/3.3V	FAN/opposite side	service life is up to 8000 hrs	Mid
Inovafitness/ SDS018 [164]	59x45x20/-	4.7 to 5.3	70	4mA/laser and fan sleep/low power operating mode	Laser sleep	1/10	UART,PWM/3.3V	FAN/same side	service life is up to 8000 hrs	Mid
NanoSense/ PM2036 [163]	36x36x23.8/ 17.5	5	80	0.4mA	No	6/-	I2C/3.3V	FAN	>1y	NA
Panasonic/ LED Type PM2.5 Sensor [147]	45×22×52/-	5	100	NA	No	1/-	I2C/-	/same side	NA	NA
Panasonic/ SN-GCJA5<br><br>Laser Type PM Sensor [165]	37x37x12/-	5	100	NA	No	1/28	I2C,UART/-	FAN	5y	Mid
Plantower/ PMS 1003/ [80] [131]	65×42×23/-	5 to 5.5	100	<1mA/<br><br>adaptative acquisition frequency	No	1-10/-	UART/3.3V	FAN/opposite side	MTTF ≥3y	Mid

Plantower/ PMS 7003/ [55] [81] [82] [124] [129]	48x37x12/-	5 to 5.5	100	<1mA/ adaptative acquisition frequency	ON/OFF	1-10/-	UART /3.3V	FAN/same side	MTTF ≥3y	Mid
Plantower/ PMS A003/ [76] [125]	35x38x12/-	5 to 5.5	100	<1mA/ adaptative acquisition frequency	No	1-10/-	UART/3.3V	FAN/same side	MTTF ≥3 Year	Mid
SAMYOUNG S&C/ PSML [148]	59x45x17/25	5	85	NA	NA	-/60	LPO(Low Pulse Occupancy)/ Vcc	Heater	5y min	NA
SAMYOUNG S&C/ PSMU [148]	59x45x17/25	5	85	NA	NA	-/60	UART/-	Heater	5y min	NA
Sensirion/ SPS30/ [118] [132]	40.6x40.6x12.2/26	4.5 to 5.5	80	<50µA/sleep -mode - idle-mode	NA	1/30	UART, I2C/-	FAN	>10y/Maximum long-term number concentration precision limit drift2 0 to 1000 #/cm³ ±12.5 #/cm³ / y 1000 to 3000 #/cm³ ±1.25 % m.v. / y	High
Sharp/ DN7C3CA007 [166]	53x51x40/53	5	105	NO	EXT	0.01/-	Analog/3.4V	FAN/opposite side	15000hour MTTF/Laser diode:50% degradation/5 y	Mid

Sharp/ GP2Y1010AU0F/ [63] [65] [67] [83] [134] [135] [136]	46x30x17.6/16	5	40	NO	EXT	0.001/-	Analog/-	NO/opposite side	Laser diode:50% degradation/5 years	Low
Shinyei/ PMS1/ [93] [137]	71.4x76.4x36.7/130	12	380	NA	NA	NA	Ethernet/-	Heater	NA	NA
Shinyei/ PPD20V/ [138] [139]	88×60×20/38	5	160	NA	No	-/60	PWM/-	Heater	7y	NA
Shinyei/ PPD42NJ/ [63] [138] [140]	59×45×22/24	5	90	NA	No	-/60	PWM/-	Auto suction by a built-in heater resistor	7y	NA
Shinyei/ PPD60PV-T2/ [138] [141]	88×60×22/-	5	140	NA	No	-/60	PWM/-	Heater	3y	NA
Shinyei/ PPD71 [149]	34x30x28/-	5	100	NA	NA	-/60	UART/-	Auto suction by a built-in heater resistor	7y	NA
TianjinFigaro-isweek/ TF-LP01 [167]	51x33x22/-	5	100	NA	NA	10/-	UART, PWM/-	FAN	NA	Mid
Winsen/ ZH03/ZH03A/ZH03B/ [67] [82] [142]	50x32.4x21/-	5	120	<10mA	NA	-/45	PWM/-	FAN/opposite site	3y in the air	Mid
Winsen/ ZH06- I [168]	47×37×12.2/-	4.9 to 5.5	120	<20mA	NA	-/45	UART, PWM/-	FAN/same side	NA	Low

Winsen/ ZPH01 [150]	59.47×44.5×20/-	5	150	NA	NA	-/300	PWM,UART/-	Heater	NA	Low
YAGUCHI ELECTRIC CORP./ SDS021 [169]	42.5x32x24.5/-	5	70	10mA	No	1/-	UART,PWM/-	FAN	NA	NA
